# Micrometer-Scale Graphene-Based Liquid Cells of Highly Concentrated Salt Solutions for In Situ Liquid-Cell Transmission Electron Microscopy

**DOI:** 10.1021/acsomega.4c05477

**Published:** 2024-09-12

**Authors:** Yuga Yashima, Tomoya Yamazaki, Yuki Kimura

**Affiliations:** Institute of Low Temperature Science, Hokkaido University, Kita-19, Nishi-8, Kita-ku, Sapporo 060-0819, Japan

## Abstract

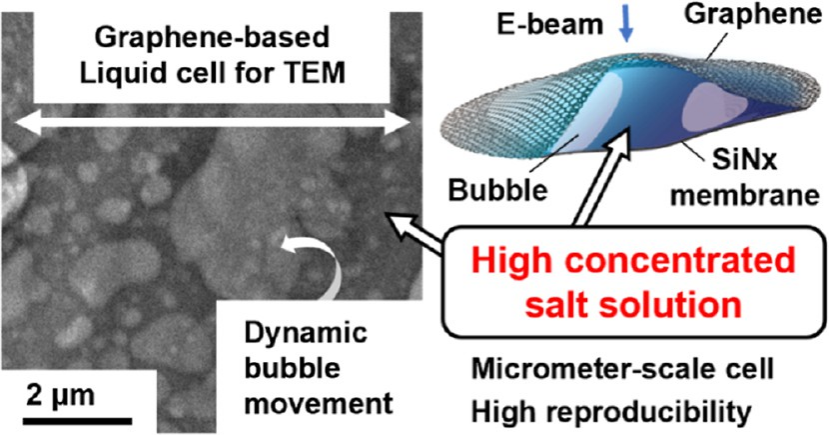

In situ liquid-cell transmission microscopy has attracted much attention as a method for the direct observations of the dynamics of soft matter. A graphene liquid cell (GLC) has previously been investigated as an alternative to a conventional SiN_*x*_ liquid cell. Although GLCs are capable of scavenging radicals and providing high spatial resolutions, their production is fundamentally stochastic, and a significant compositional change in liquids encapsulated in GLCs has recently been pointed out. We found that graphene-based liquid cells were formed in nano- to micrometer sizes with high reproducibility when the concentration of the encapsulated aqueous salt solution was high. In contrast, when we revisited conventional fabrication methods, water-encapsulated GLC was formed with low yield, and any electron diffraction spots from ice were not confirmed by a cooling experiment. The reason for this was the presence of intrinsic defects in the graphene, the presence of which we confirmed by the etch-pit method. The shrinkage of a water-encapsulated cell and a decrease in the bubble area in an aqueous (NH_4_)_2_SO_4_ solution cell suggested that volatile water molecules and gas molecules can leak from the cells during the fabrication and observation processes. Further revision of the conditions for the formation of liquid cells and a reduction in the number of intrinsic graphene defects are expected to lead to the provision of graphene-based liquid cells capable of encapsulating dilute aqueous solutions or pure water.

## Introduction

The direct observation of liquid samples by transmission electron microscopy (TEM) is a powerful way to understand various phenomena in the field of crystal growth, such as nucleation, crystallization, dissolution, movement of particles, or bubbling. Many researchers have shown interest in the microscopic dynamics of these phenomena at scales as small as the nanometer range.^1−3^ TEM requires specimens under vacuum conditions because of the use of an electron beam as a probe. Consequently, thin films capable of transmitting an electron beam must be used to prevent the evaporation of the liquid sample into the TEM chamber.

The use of an amorphous silicon nitride (SiN_*x*_) film permits the observation of many interesting phenomena in liquid by using TEM.^4−7^ Although it is commercially well developed, this technique has some experimental limitations in achieving sufficiently high spatial resolutions to capture microscopic phenomena. SiN_*x*_ liquid cells bulge due to the high vacuum present in the TEM chamber, and the observable area of appropriate thickness is limited to the edge of the SiN_*x*_ window.^8^ Additional pumping systems should be used to suppress the bulge by applying negative pressure at the outlet.^9,10^ Also, the general thickness of commercial SiN_*x*_ films is about 50 nm. Decreasing the thickness requires a decrease in the observable area of the film or more-technical development to avoid film breakage.^11^

Graphene, which consists of a planar hexagonal network of carbon atoms, is an alternative material for thin films for liquid cells. In 2012, Yuk and co-workers proposed a graphene liquid cell (GLC) as an alternative to the conventional SiN_*x*_ liquid cell.^12^ A GLC can be fabricated by encapsulating a liquid between two sheets of graphene or between a sheet of graphene and another substrate such as a SiN_*x*_ membrane.^13−15^ Graphene has a high transparency to electrons and is mechanically tough; moreover, it is capable of scavenging radicals formed through radiolysis by the electron beam. The factors permit the achievement of higher spatial resolutions than those attainable with a SiN_*x*_ liquid cell.^16^ Furthermore, graphene has attracted particular attention as a soft-matter encapsulation material for liquid cell-TEM (LC-TEM).^17,18^

Although GLCs have considerable advantages for the direct observation of targets in liquids at atomic resolutions, they require a careful examination of the components of the liquid that are encapsulated in the GLC. It is necessary to take care to verify the presence of water in a GLC, and the most reliable method for doing so is to check for the presence of a water excitation peak by electron energy-loss spectroscopy (EELS).^19^ An EELS study recently pointed out the solute concentration effect of GLCs.^20^ Aqueous CeCl_3_ solution encapsulated in the GLC becomes significantly concentrated, with a cerium-to-oxygen ratio approaching that of the hydrated salt CeCl_3_·7H_2_O.

The aim of our research was to explore the microscopic dynamics of a dilute aqueous solution by direct observation. Our representative target was to capture the nucleation and crystallization of ice from pure liquid water or from a dilute solution. Although the formation of cubic ice in a GLC has been reported,^21^ Zhou et al. have suggested that this might have been due to salt contamination.^22^ It has been recently shown, however, that cubic ice does form on a TiO_2_ crystal in a GLC; in this case, the TiO_2_ crystal acts as a scavenger for generated radicals.^23^ Although an ice Ih fraction has been captured from its oxygen peak in EELS,^24^ to the best of our knowledge there has been no report of ice Ih formation in a GLC.

In this study, we revisited the previously reported method for the fabrication of GLCs with the aim of fabricating those of pure water free of contamination originating from the etchant used in the production of the graphene sheet. Unlike the case of water, GLCs of concentrated etchant and solutions of similar composition form at a micrometer scale, which is larger than that of previously reported GLCs. The observation of a decrease in the bubble area in a GLC and an etch-pit experiment suggested to us that focusing on defects in the graphene might be a key to exploiting GLCs in studies on the chemistry of dilute solutions and pure water.

## Results and Discussion

### Attempting a Fabrication of GLCs with Pure Water by the Conventional Method

**Figure 1:**
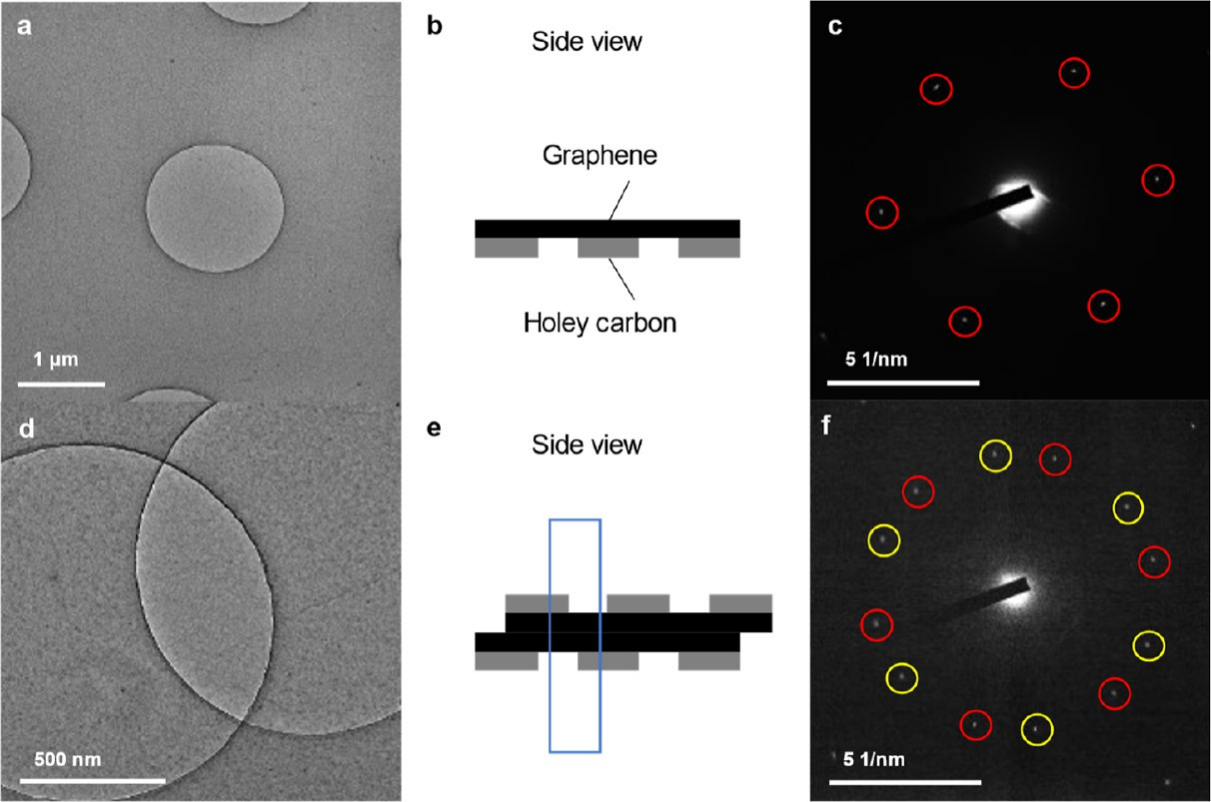
Attempts to form a GLC by the conventional method. (a) A bright-field TEM image of a holey carbon grid on which graphene was transferred. (b) Schematic illustration of a side view of (a). (c) SAED pattern acquired from the holey area of (a): a 6-fold symmetric pattern is highlighted by the red circles. (d) Bright-field TEM image of a GLC without water fabricated by the conventional method. The grids were fabricated by placing two graphene sheets facing each other and attempting to sandwich droplets of pure water (Supporting Information; Figure S1a). (e) Schematic illustration of a side view of (d); the region in the blue dotted square corresponds to the area of (d). (f) SAED pattern acquired from the crossing area of d: two sets of 6-fold symmetric patterns are highlighted by the red and yellow circles.

Most previously reported GLCs were fabricated by encapsulating liquid between two sheets of graphene.^25^ Following this method, we transferred a sheet of graphene onto a holey carbon TEM grid (Figure 1a,b), and we confirmed the presence of a 6-fold symmetry in the selected area electron diffraction (SAED) pattern from a holey area (Figure 1c), confirming that the graphene had been successfully suspended on the TEM at a micrometer scale without any folding or wrinkling. An attempt was made to fabricate a GLC of pure water by placing a drop of pure water on the graphene sheet and sandwiching it with a second graphene sheet on a holey carbon TEM grid [Figure 1d,e, and Supporting Information Figure S1a]. We expected that a strong contrast due to the encapsulated water between the two sheets of graphene would appear but, in fact, no such contrast could be observed.

It is well-known that water readily undergoes radiolysis on electron irradiation, producing species such as hydrated electrons (e_h_^–^), H, OH, H_3_O^+^, and HO_2_, resulting in the production of hydrogen and oxygen molecules.^26^ In the present study, no bubbles were formed, even on increasing the electron dosage. To confirm the presence of graphene itself, the SAED pattern (Figure 1f) was recorded from the area where the two circles intersected in Figure 1d. Each of the two circles in Figure 1d corresponds to a holey area of one of the TEM grids, meaning that only a layer of graphene was expected to be present. The SAED pattern clearly showed two sets of 6-fold symmetries (the red and yellow circles in Figure 1f), suggesting that two sheets of graphene faced each in a planar manner without encapsulating any water. A low mag image of the TEM grid (Supporting Information; Figure S2) excludes a gradual change in contrast that was not significant in Figure 1d. We attempted this procedure more than ten times, each time observing the entire TEM grid, but we did not observe any encapsulation of water.

### Fabrication of GLCs by the “Free-Standing” Method

**Figure 2:**
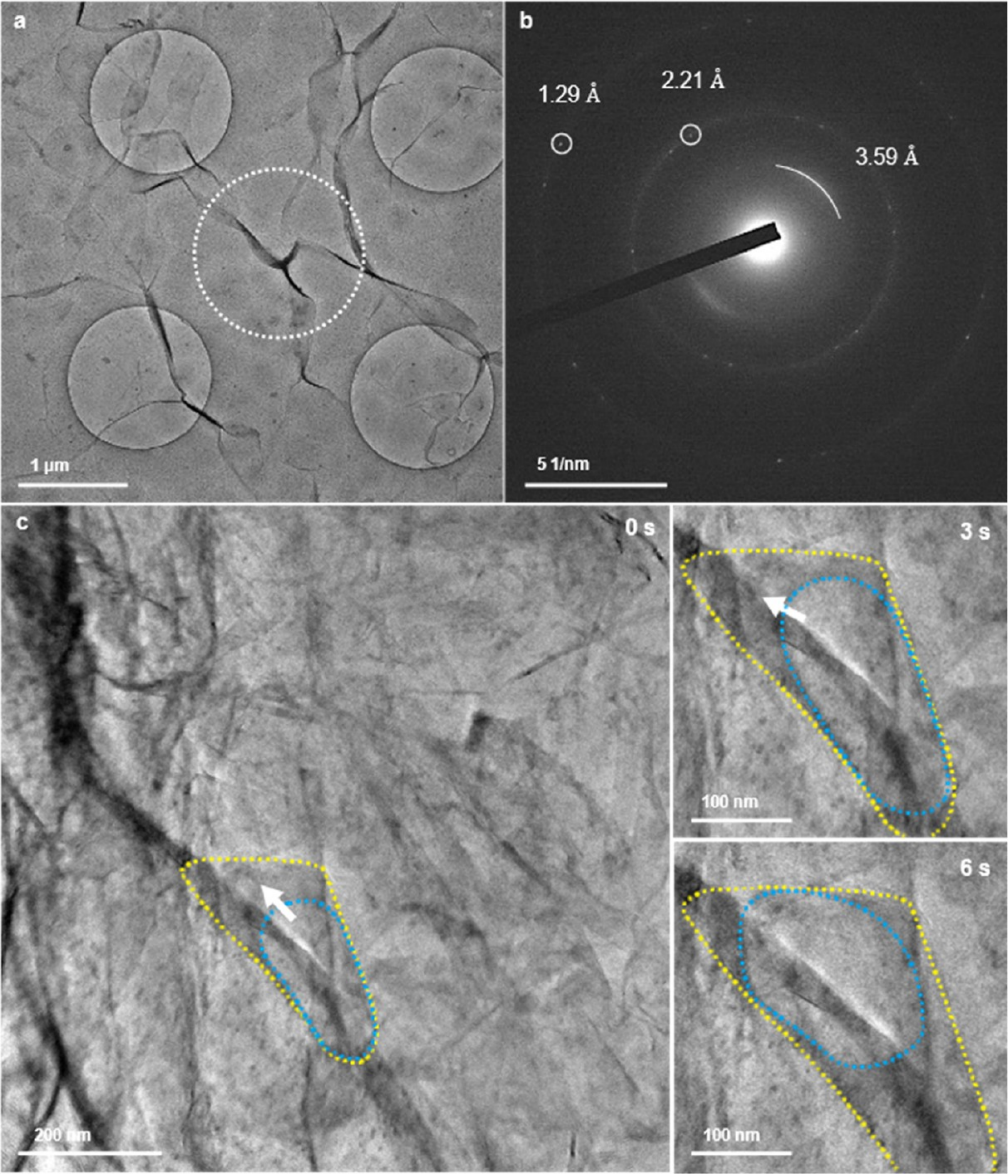
Attempts to form a GLC by the free-standing method. The grids were fabricated by the method shown in Supporting Information, Figure S1b. (a) Bright-field TEM image of a holey carbon grid with a free-standing graphene sheet, observed at 80 K. (b) SAED pattern acquired from the dotted circle area in (a). (c) Snapshots of the bright-field TEM images of a GLC in folded graphene encapsulating displaced water from the etching solution at 0, 3, and 6 s. The yellow and blue dotted lines are guides for the GLC and the bubble inside it, respectively. The direction of movement of the bubble is indicated by white arrows. The full video is available as Supporting Information Video S1.

To facilitate the encapsulation of water, we modified the method for fabricating the GLCs by introducing “free-standing” graphene on the surface of the water. In this context, “free-standing” means not being supported by any substrate, such as a carbon TEM grid, before encapsulation. We hoped that the “free-standing” condition would allow the graphene to bend and wrinkle to wrap around the water. In this case, the free-standing graphene floating on the water was directly scooped onto a holey-carbon TEM grid on which graphene had already been transferred (Supporting Information; Figure S1b). After fabrication, the grid was observed at 80 K in the hope of easier detection of the water from diffraction contrasts (Figure 2a). The solubility in water of gas molecules generated by radiolysis increases at lower temperatures, a process that is thought to suppress the formation of bubbles. The darker contrasts that could be identified as bending, wrinkling, or folding of the planar sheet of graphene were spread over the whole carbon grid, unlike those in Figure 1d, in which both sheets of graphene were transferred onto the grid in advance. Circular dark contrasts of approximately 100–200 nm in diameter were also distributed across the grid.

To confirm the encapsulation of water in the strong contrast area, a SAED pattern (Figure 2b) was acquired from the area defined by the dotted circle in Figure 2a. The pattern consisted of 6-fold symmetric patterns at 1.29 and 2.21 Å from graphene, and a blurry halo-like diffraction at 3.59 Å. It has previously been shown that the van der Waals thickness of two to four layers of graphene is 3.70 Å and that the (0 0 2) spacing of graphite shrinks to 3.36 Å.^27^ This is in good agreement with our result, because we used thicker graphene with six to eight layers for the fabrication of GLCs by our “free-standing” method. No diffractions associated with crystalline or amorphous ice were observed. Even after the specimen was warmed to room temperature, the TEM contrast did not change significantly, and no emergence of bubbles was observed on increasing the electron-beam intensity, suggesting that water was not encapsulated in this area. The circular contrasts in Figure 2a could be any contamination or oxidation of the graphene due to the overnight free-standing treatment of graphene on solutions during fabrication, requiring further elemental analysis.

Although most of our experiments with pure water resulted in no crumpling of the graphene (Figure 1d) or no encapsulation of any liquid (Figure 2a), a few GLCs with bubbles that resembled those previously were found on the holey carbon TEM grid (Figure 2c). Free-standing graphene on water was scooped by the holey carbon TEM grid (Supporting Information; Figure S1b). The holey carbon sheet on which we planned to transfer the graphene transferred was partially broken for some mechanical reason during the procedure. As a result, this permitted the graphene to fold and roll up, with encapsulation of the solution. A bubble which had a brighter contrast (the blue dotted line in Figure 2c) existed inside the strip-shaped GLC shown by the yellow dotted line in Figure 2c. The bubble could have been produced by radiolysis of the liquid in the GLC or precipitation of dissolved gases. Under electron-beam irradiation, the bubble moved upward inside the GLC during 0–6 s (Figure 2c), and then gradually expanded downward after 28 s, following an increase in the electron dose rate at 26 s, suggesting the presence of liquid around the bubble (Supporting Information; Video S1). Although cooling experiments were conducted for easier detection of water as ice, the presence of GLC-encapsulated water displaced from the etching solution could be confirmed from the bubble’s movement at room temperature without the cooling. The displaced water was encapsulated by the accidental folding and rolling up of graphene, but this technique cannot be used for the controlled production of water-encapsulated GLCs.

### Attempts to Form a Graphene–SiN_*x*_ Liquid Cell by a Free-Standing Method

Some attempts have been made to produce GLCs with a controlled geometry by introducing a spacer between two sheets of graphene^28,29^ or by obtaining a certain volume with a microwell grid.^15,30^ We designed a graphene–SiN_*x*_ liquid cell (GSLC) (Supporting Information; Figures S1c–e) with a SiN_*x*_ microwell TEM grid (Norcada Inc., Edmonton; Supporting Information Figure S3). The grid had circular microwells of diameters 1, 2, or 3 μm and a depth of 60 nm, permitting the formation of a volume for a GSLC along the wall of the microwell. Graphene was used on one side only in the GSLCs to make it easier to discuss the ability of graphene to encapsulate liquids, water-leak-proof SiN_*x*_ membrane on the other side.

**Figure 3:**
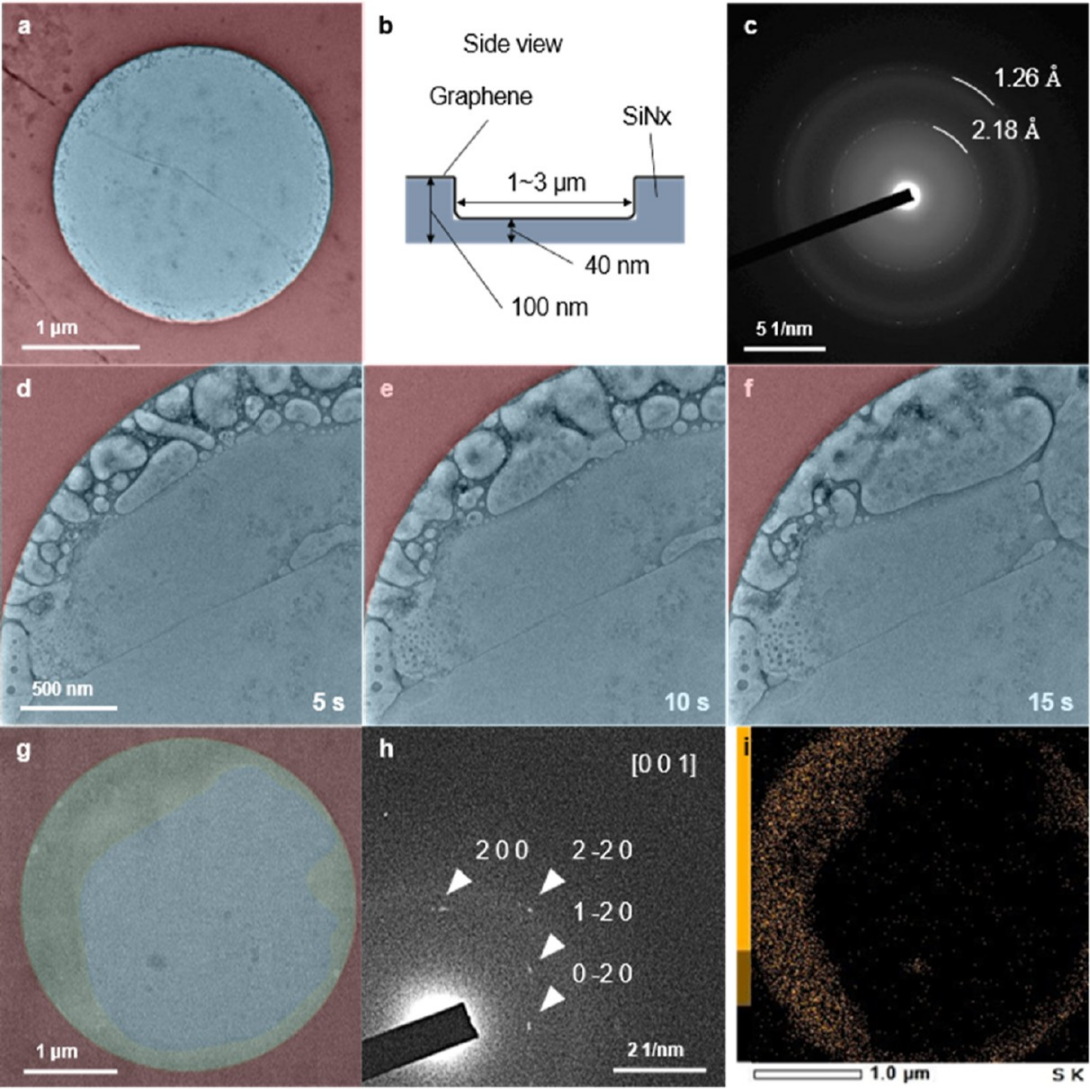
Attempts to form a GSLC encapsulating water. The grid was fabricated by the method shown in Supporting Information, Figure S1c. (a) Bright-field TEM image of GSLCs after attempts to encapsulate displaced water from the etching solution. The circular area (blue) and the surrounding area (red) had thicknesses of 40 and 100 nm, respectively. (b) Schematic illustration of a side view of (a). (c) SAED pattern acquired from the blue area of (a). (d–f), Snapshots of the bright-field TEM image of the GSLC showing the formation and movement of bubbles at 5, 10, and 15 s. The full video is available as Supporting Information Video S2. (g) Bright-field TEM image of a large-area GSLC on a SiN_*x*_ microwell grid cooled at 253.8 K. The frozen GSLC is highlighted in yellow. (h) the corresponding SAED pattern for the GSLC in (g). The diffraction spots (white arrowheads) were attributed to crystalline ammonium sulfate from [001] (AMCSD code: 0012987). (i) STEM–EDS mapping of sulfur corresponding to (g). The mapping is rotated 20° counterclockwise relative to (g). See Supporting Information, Figure S4 for the full EDS images and the spectrum.

Free-standing graphene floating on the water displaced from the etching solution was directly scooped up by the microwell grid (Figure 3a,b and Supporting Information, Figure S1c). No bubble formation, which indicates the presence of water, was found in the grid; only graphene wrinkles crossing the image were observed. Those signatures remained unchanged and no bubbles were observed, even on increasing the intensity of the electron beam. This result is similar to that obtained by using the conventional and the free-standing fabrication methods. SAED patterns (Figure 3c) acquired from the well area (Figure 3a) showed the diffraction pattern of graphene and a hollow pattern from the SiN_*x*_ membrane at the bottom of the microwell grid (Supporting Information; Figure S3).

Encapsulation of a liquid in a GSLC with bubbles was observed only once in nine similar experiments (Figure 3d–f and Supporting Information; Video S2). The bubbles produced as a result of radiolysis moved around and merged along the microwell. After repeating these experiments for the GSLCs, we succeeded in fabricating a second GSLC along the wall of the microwell (Figure 3g). The GSLC was cooled to 253.8 K with a Peltier-cooled TEM holder.^31^ A strong contrast (the yellow area in Figure 3g) confirmed a distribution of the GSLC along the 60 nm-high round-shape wall (the red area in Figure 3g). An acquired SAED pattern from the yellow area (Figure 3h) did not correspond to water ice but, instead, was attributed to the [0 1 0] SAED pattern of crystalline ammonium sulfate (AMCSD code: 0012987). Bubble formation due to radiolysis was suppressed by an increase in the solubility of the gas molecules due to the cooling, permitting elemental mapping by energy-dispersive X-ray spectroscopy–scanning transmission electron microscopy (EDS–STEM). This mapping showed a broad distribution of not only oxygen, but also sulfur, along the microwell (Figure 3i; see Supporting Information; Figure S4 for a full EDS mapping and the corresponding spectrum). No SAED signals from ice were acquired at any stage of the cooling experiment. This does not exclude the possibility of ice crystallization at temperatures lower than 253.8 K, i.e., the presence of water could be allowed due to unique thermodynamics of water trapped between the thin films. Further cooling experiments with lower freezing temperatures are needed to discuss the presence of water in the GSLC. Although, the presence of a significant amount of crystalline ammonium sulfate, even after the etching solution had been carefully displaced by pure water, prompted us to carefully examine the compositions of the encapsulated liquids in the GLC (Figure 2c) and GSLC (Figure 3d).

### Encapsulation of Highly Concentrated Aqueous Solutions in GSLCs with High Reproducibility

**Figure 4:**
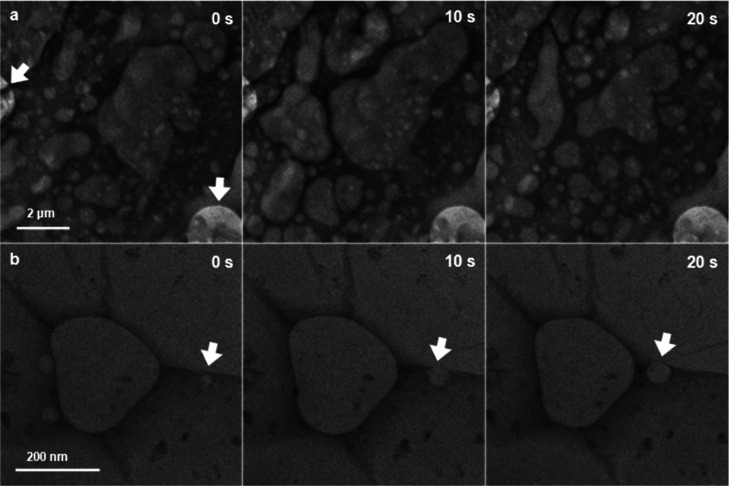
Fabricated GSLCs at a micrometer-to-submicrometer scale. (a) Series of bright-field TEM images of a GSLC of an etching solution on a SiN_*x*_ microwell grid at 0, 10, and 20 s. The full video is available as Supporting Information Video S3. The 60 nm-deep microwells are indicated by white arrows. The grid was fabricated by the method shown in the Supporting Information, Figure S1d. (b) A series of bright-field TEM images of a GSLC of a 0.4 M aqueous (NH_4_)_2_SO_4_ solution in a SiN_*x*_ microwell at 0, 10, and 20 s. The bubble tracked in Supporting Information, Figure S5b is indicated by white arrows. The full video is available as Supporting Information Video S4. The grid was fabricated by the method shown in Supporting Information, Figure S1e.

Next, we examined direct scooping by the SiN_*x*_ microwell TEM grid of the etching solution under free-standing graphene to fabricate GSLCs of the etching solution (Supporting Information; Figure S1d). As a result, we observed a successful encapsulation of the etching solution in the GSLCs with a high reproducibility (Figure 4a). The GSLCs even spread out over the area of a 100 nm-thick SiN_*x*_ membrane, although we initially intended to encapsulate the etching solution inside the circular SiN_*x*_ microwell (the white arrow in Figure 4a), which shows a brighter contrast due to the lower thickness (40 nm) of the SiN_*x*_ membrane. It was easily confirmed from snapshots of the GSLCs at 0, 10, and 20 s that the GSLC had an area significantly larger than 10 μm^2^. The area was roughly assessed from the extent to which bubbles moved around (see Supporting Information, Video S3 for the full time range). Those bubbles were considered to have emerged and moved as a result of irradiation by the electron beam. Those GSLCs of a micrometer scale were distributed all over the TEM grid, as was confirmed by a follow-up experiment. This is completely contrary to the results of experiments involving the encapsulation of pure water (Figures 1 and 2), which resulted in no formation of GLCs or a stochastic rare formation of GLCs in a few parts of the TEM grids.

The broad distribution of GSLCs with high reproducibility was also confirmed for an aqueous solution of (NH_4_)_2_SO_4_ of similar composition and concentration to the etching solution [10 w/v % (0.43 M) (NH_4_)_2_S_2_O_8_]. Etching solution under floating graphene was carefully replaced by a 0.4 M aqueous solution of (NH_4_)_2_SO_4_. The high stability of the graphene flake after the displacement enabled us to scoop the liquid with the graphene onto the SiN_*x*_ microwell TEM grid (Supporting Information; Figure S1e). Fabricated GSLCs were distributed throughout the TEM grid, as in the case of the etching solution in Figure 4a. One of those GSLCs present in a SiN_*x*_ microwell had a dendritic shape (Figure 4b). From the snapshots for 20 s, we were able to confirm the movement and merging of some surrounding bubbles to form a large bubble at the center of the dendritic GSLC. A video recording showed that the GSLC was stable for up to 40 s (see Supporting Information Video S4 for the full time range). The formation of micrometer-to-submicrometer-scale GSLCs enabled us to conduct an additional analysis of the encapsulated bubbles, showing more than two orders of range in the velocities (Supporting Information; Figures S5–S9). Note that the concentration of the encapsulated liquids in Figure 4 was significantly higher, at about 0.4 M, and this could drive the formation of GSLCs with a high reproducibility.

### Bubbling Behaviors of Dried Highly Concentrated Aqueous Solutions

**Figure 5:**
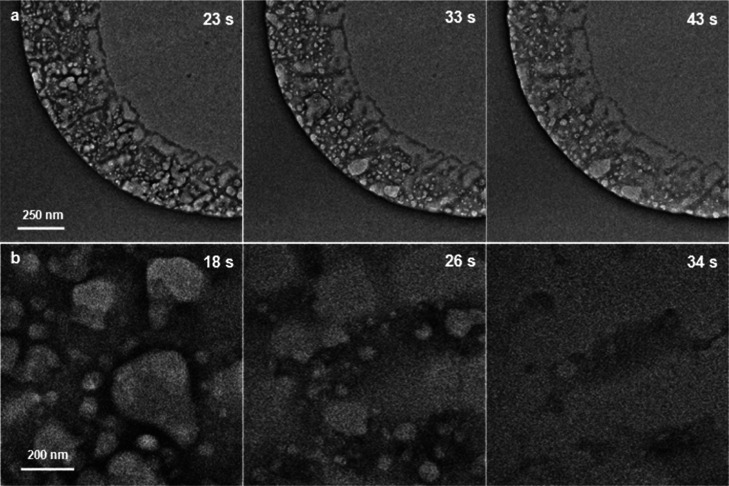
Bubbling and liquid-like behavior of solutions in an open system. (a) Series of bright-field TEM images of ten-times-diluted etching solution at 23, 33, and 43 s. The full video is available as Supporting Information Video S5. (b) Series of bright-field TEM images of 0.4 M aqueous (NH_4_)_2_SO_4_ solution at 18, 26, and 34 s. The solution was simply dropped onto a SiN_*x*_ microwell TEM grid without the encapsulation by graphene. The full video is available as Supporting Information Video S6.

The formation of the GSLCs shown in Figure 3d–f might be due to the presence of residual etching solution inside the microwell, even after five cycles of repeated displacement with pure water. It was confirmed that the etching solution tends to remain on the TEM grid due to its low vapor pressure. The etching solution was diluted ten times and dropped onto a SiN_*x*_ microwell TEM grid. The grid was directly loaded into a TEM chamber without scooping free-standing graphene. Residues of the dried etching solution remained along the wall of the microwell (Figure 5a and Supporting Information Video S5) in a manner reminiscent of the previously reported ring-type GSLCs on SiN_*x*_ membranes.^32^ The residue bubbled due to radiolysis, and those bubbles coalesced with neighboring bubbles. It should be emphasized that the slight movement of bubbles during successive deformation due to the coalescence was captured even without encapsulation by graphene. Immediately after capturing the video, we conducted an elemental mapping by EDS (Supporting Information Figure S10) for the same microwell shown in Figure 5a. The area roughly in the lower left half of Supporting Information Figure S10 corresponds to the area shown in Figure 5a. This showed the presence of copper from the CVD substrate for graphene, as well as oxygen and sulfur from the etching solution.

Because the bubbling was observed for the residue after the etching solution had dried, we also confirmed that this occurred with an aqueous solution of (NH_4_)_2_SO_4_. A 0.4 M aqueous solution of (NH_4_)_2_SO_4_ was simply dropped onto a SiN_*x*_ microwell TEM grid, which was then directly loaded into a TEM chamber without encapsulation with graphene. The residue from the aqueous (NH_4_)_2_SO_4_ solution after drying also bubbled due to radiolysis, and the resulting bubbles underwent successive coalescences (Figure 5b). The contrast of those TEM images gradually decreased as a result of the bubbling and sublimation like (NH_4_)_2_SO_4_ aerosol particles which were observed in the vacuum by conventional TEM;^33^ finally, these completely disappeared due to the focusing of the electron beam (Supporting Information; Video S6). The exposed area showed a brighter contrast compared to a less exposed area, clearly showing the evaporation of the solution due to the irradiation of the electron beam (Supporting Information; Figure S11). Although those liquids [the etching solution in Figure 5a and the aqueous (NH_4_)_2_SO_4_ solution in Figure 5b] had completely dried at the end, the lifetime of the emerging bubbles were in time ranges similar to those for the GLCs shown in Figure 4. A similar liquid-like behavior of a beam-irradiated salt has been observed for dried sodium phosphate buffer solution.^34^ The temporal liquid-like behavior of a dried solution due to the movement and deformation of the bubbles, as observed in the present study, suggests that caution should be exercised in discussing studies using GLCs (or GSLCs) solely on the basis of physical phenomena associated with bubbles. Although the detection of a water signal by EELS is known to be an effective way of verifying the presence of water in GLCs or GSLCs,^19^ this technique, like many other verification methods, does not guarantee the presence of the molecules between the thin films. Whether or not what we are observing as GLC is really present in the encapsulated space must be comprehensively determined from whether the liquid of interest does not evaporate during the observation process (e.g., the evaporation in the case without graphene, Figure 5) and whether the bubbles generated in the liquids are in motion reflecting the constraints of the geometry (e.g., the bubble motions along the boundary between graphene and substrate adhesion, Supporting Information Videos S1, S2, S3 and S4).

### GLC Imperfections and Graphene Defects

**Figure 6:**
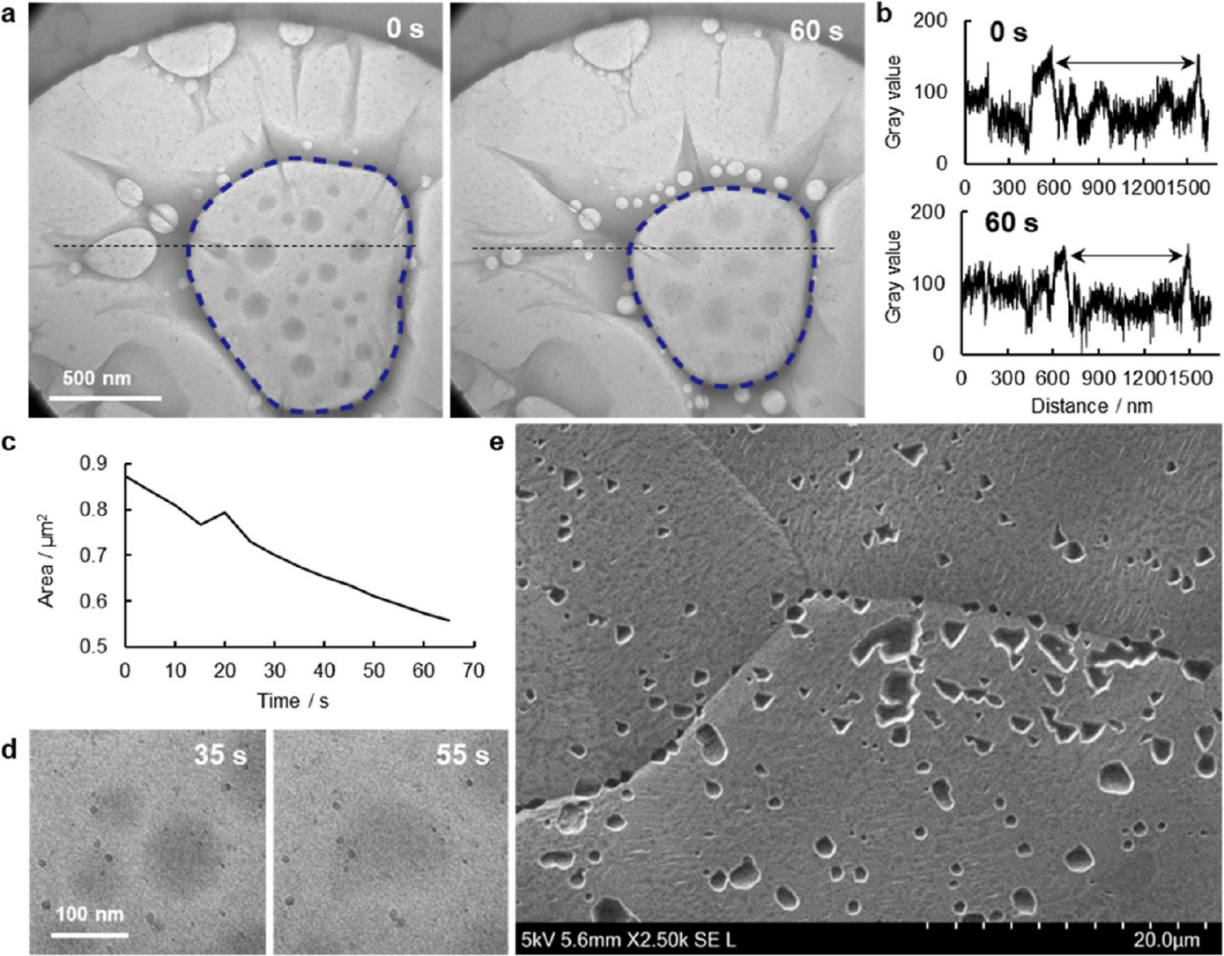
Leakage from a GSLC and defects in graphene. (a) Bright-field TEM images of a GSLC of 0.4 M aqueous (NH_4_)_2_SO_4_ solution on a SiN_*x*_ microwell grid at 0 and 60 s. The full video is available as Supporting Information Video S7. (b) Line profiles of the gray values of the black dot lines in a at 0 and 60 s. (c) Time evolution of the area of the large bubble (the blue dashed lines in a). (d) Series of enlarged views of the fusion of the inner liquid droplets in the large bubble at 35 and 55 s. (e) Secondary electron image of etch pits on a Cu foil. A 10% w/v aqueous (NH_4_)_2_S_2_O_8_ solution was dropped onto the graphene surface on the Cu foil and then the foil was rinsed with double-distilled water.

For the development of GLCs of pure water and dilute solutions, it is important to carefully discuss the ratio of water molecules to contaminants or solutes. For instance, the liquid encapsulated in a successfully fabricated GLC (Figures 2c and 3d–f) could be a dilute solution (desirably pure water) or a concentrated solution containing contaminants from the etching solution. The liquid will be a dilute solution if no leakage of water from the GLC occurs during the fabrication and observation processes. In contrast, if a significant leakage occurs, the liquids will be far-from-dilute solutions. To verify the presence of dilute solutions in GLCs (or GSLCs), an easily understood method would be to confirm the formation of ice at a low temperature. The cooling experiment at 253.8 K (Figure 3g) did not confirm the characteristic SAED patterns originating from ice crystal, indicating the presence of a highly concentrated solution of the contaminant (ammonium sulfate from the etching solution), suggesting that leakage of water from the GSLC had, indeed, occurred. Leakage of an encapsulated liquid under TEM observation was confirmed for a GSLC of a 0.4 M aqueous (NH_4_)_2_SO_4_ solution (Figure 6a and Supporting Information Video S7). The GSLC with a branch was formed at the center of the microwell at 0 s. A large bubble in a middle of this GSLC (shown by the blue dotted line in Figure 6a) was already present when the observation started, and it was found that small bubbles emerged from edges of the GSLC, and these continuously moved toward the large bubble (the blue dashed lines in Figure 6a).

Although radiolysis successively occurred under the TEM observation, the lateral length of the large bubble in the GSLC was found to gradually decrease (Figure 6b). From the line profiles of the black dotted lines in Figure 6a, the lateral length was found to decrease by about 200 nm in the 60 s after the start of the acquisition of the video. An image analysis showed that the area of the bubble decreased by more than 0.3 μm^2^ (Figure 6c). This suggests a leakage of gas molecules produced by radiolysis from the GSLC into the TEM vacuum chamber. After the video had been captured, SAED patterns were immediately acquired to confirm the presence of graphene around the GSLC (Supporting Information; Figure S12). Unlike the SAED pattern from the area near the GSLC, no signal of graphene was observed from the middle of the GSLC. It could be that the graphene on the large bubble in the middle bulged as a result of the increase in the circularity of the bubble. Also, the presence of round-shape strong contrasts was confirmed inside the bubble area (Figure 6d). These contrasts could arise from liquid on the SiN_*x*_ membrane. The fact that this was deformed while sticking together during the observation supports the view that the graphene was not in contact with the substrate in the central part of the big bubble. Movements on the SiN_*x*_ membrane were observed for about 1 min, suggesting that the bubble in the GSLC was not completely open to the vacuum chamber, and this allowed bubbles to move and coalesce without immediate evaporation.

A potential explanation that links the present results, the low yield of the GLCs with pure water, and the decrease in the bubble area in the GSLC, is the presence of defects in graphene through which volatile molecules preferentially permeate and evaporate in preference to other molecules or ions. Graphene is theoretically an impermeable material,^35,36^ and this fact motivated the development of GLCs. In contrast, as mentioned in the context of crystallization of CaCO_3_ in GLCs,^37^ defects in graphene cannot be ignored in experimental studies. When added to knock-on damage at the atomic scale under TEM observation, intrinsic defects at the nanometer or larger scale, through which volatile molecules can easily permeate, are important when we are considering leakages, even in the cell-fabrication process before TEM observation. Leakage of water from GSLCs under ambient conditions was simply confirmed by optical microscopy (Supporting Information; Figure S13). Free-standing graphene floating on supercooled double-distilled water at −5 °C was scooped by a hydrophilically pretreated SiN_*x*_ microwell TEM grid. The water was supercooled to increase its viscosity^38^ for better encapsulation in GSLCs. Immediately after the cell fabrication, encapsulation of water in the middle area of the grid was confirmed by a change in the reflection of light due to bulging of the graphene. On standing overnight at −5 °C, a loss of majority of water or ice (i.e., less bulging of graphene) in the middle area was confirmed. At that stage, we could not exclude the possibility that water molecules leaked, not only through intrinsic defects in the free-standing graphene, but also through spaces between the graphene and the SiN_*x*_ substrate and/or cracks in graphene caused by the scooping of free-standing graphene. The TEM image of the GSLC only showed a distribution of circular dark contrasts of approximately 100–200 nm in diameter which are similar to that of Figure 2a. The contrasts did not change and no bubble emergences were observed, suggesting any contamination or oxidation of the graphene due to the overnight free-standing treatment of graphene on etching solution and on the supercooled water. Then, the presence of intrinsic defects in the graphene used in the present study was directly confirmed by the etch-pit method.^39^ An etching solution was dropped on the surface of graphene produced by CVD on a copper foil. If the graphene was defective, the etching solution would be able to attack the copper through the defects to form etch pits. Scanning electron microscopy (SEM) showed that etch-pits were indeed formed at a density of 0.1 etch pit/μm^2^ (Figure 6e). Although it was difficult to estimate the sizes of defects from the resulting etch pits, this experiment clearly showed that the graphene contained defects through which water molecules in the etching solution could permeate. CVD-grown graphene on a metal (as represented by copper foil), which is known to contain defects, has been used in most previous studies on GLCs.^40^ Even for sizes smaller than the observed etch pits density, water–GLC yields were low and other causes of water leakage, such as reduced adhesion due to graphene surface contamination, need to be examined in the future.

The significance of the GSLCs in Figure 4 is the encapsulated area (volume) of liquid that covered the microwells on a micrometer scale. In a previous study, the highest population in the size distribution of GLCs was 100–150 nm.^41^ Also, round-shaped pocket GLCs with the highest count of 50–100 nm in pocket diameters have been fabricated.^42^ In such small GLCs, effects of confinement are possible. By introducing saturated aqueous NaCl into GLCs, it has been reported that, as a result of confinement, NaCl crystals with a hexagonal morphology are formed via a graphite-like intermediate phase, which differed from the result of a control experiment with a conventional SiN_*x*_ liquid cell.^43^ The presence of an EELS plasmon peak of water indicated that the internal pressure in GLCs is as high as 400 MPa.^44^ An atomic-force microscopy study also indicated a high inner pressure of 1–63 MPa.^45^ Although the science with the confined geometry is itself quite intriguing, it is difficult to directly apply those results to those obtained at ambient pressures. The internal pressure of the GSLCs in Figure 4a could be lower due to the weaker van der Waals interaction between the graphene and the SiN_*x*_ substrate. The present micrometer-scale GSLCs have the potential for use in studies on bulk phenomena that are not constrained by the pressure and confined space present in conventional GLCs; when combined with techniques to seal defects in graphene, such as atomic-layer deposition,^46^ GSLCs could be used for observing dilute aqueous solutions, or even water, in controllable geometries.

## Conclusion

We have attempted to develop a graphene-based liquid cell to capture the nucleation dynamics of water to ice with a higher spatial resolution than that achievable by conventional methods. First, we followed the previously reported method for the fabrication of GLCs with pure water. A planar suspension of graphene on the TEM grids resulted in no encapsulation of water. We therefore introduced “free-standing” graphene to facilitate bending and wrinkling of the graphene sheet. Some of the fabricated strip-shaped GLCs showed movements of bubbles, as previously reported. We were able to improve the geometry and volume of the encapsulation by introducing SiN_*x*_ microwell TEM grids, although only after repeated experiments. We expected the formation of water ice on cooling to 253.8 K; however, no ice was found; instead, we found ammonium sulfate, probably from the etching solution. From this unintended crystallization of the etching solution, we found that encapsulation of the etching solution occurred in the GSLC with a high reproducibility. It is noteworthy that the area of the GSLCs sometimes reached a micrometer scale. The formation of large-area GSLC was also confirmed with an aqueous (NH_4_)_2_SO_4_ solution of similar concentration. The high stability of the resulting GSLCs allowed us to study the bubble dynamics in seconds.

Having confirmed the bubbling of dried high-concentration aqueous salt solution, we were motivated to carefully consider whether or not water molecules of encapsulated liquids in “successfully fabricated” GLCs or GSLCs had leaked. The decrease in the bubble area in the GSLC of a (NH_4_)_2_SO_4_ solution showed that leakage of gas molecules occurred under the TEM observation conditions. This study highlighted the role of the etching solution of graphene, which has been less-well discussed or ignored as a mere contaminant in previous studies on GLCs. The high reproducibility of the GSLC of the etching solution at the micrometer scale was linked to the low yield of that of pure water due to leakage of water and gas molecules. Further experiments with different salt compositions and with lower freezing temperatures will be needed to verify the conditions of such liquids encapsulated in GLCs and GSLCs. If the leakage pathways could be blocked, GSLCs could become powerful tools for elucidating the dynamics of dilute aqueous solution, including water nucleation to ice, under more-ambient pressures.

## Methods

### SiN_*x*_ Microwell TEM Grid

The SiN_*x*_ microwell TEM grid (Norcada, Inc., Edmonton; Supporting Information Figure S3) has four square windows. Each window contains 81 microwells (9 × 9) of three different diameters (1, 2, and 3 μm). Each microwell is 60 nm deep between the SiN_*x*_ membranes of 100 and 40 nm thickness. All grids were subjected to a hydrophilic treatment by plasma-ion bombardment to facilitate the spreading of the target liquid on the SiN_*x*_ membrane.

### Fabrication of GLCs by the Conventional Method

For the fabrication of the GLCs with graphene supported by the holey carbon TEM grid (Supporting Information; Figure S1a), graphene (6–8 layers CVD on a Cu foil; ACS Material, LLC; Pasadena, CA) was cut into about 1 cm-square pieces with a box cutter. The graphene on Cu was sandwiched between two sheets of powder paper and flattened by pressing with a glass slide. A holey carbon TEM grid (Quantifoil Micro Tools GmbH, Groβlöbichau, Germany) was placed on the Cu foil with the graphene facing the carbon-coated side. Isopropyl alcohol (IPA) was dropped onto the Cu foil to facilitate the adhesion of the carbon sheet to the graphene.^47^ After evaporation of the IPA for 30 min, the Cu foil with the TEM grid was carefully floated on 10% w/v aqueous (NH_4_)_2_S_2_O_8_ (the etching solution which is commonly used for GLC’s fabrication) so that the Cu foil was in contact with the surface of the etching solution. After overnight contact, it was confirmed that the Cu foil had been completely etched, and the TEM grid remained suspended on a sheet of graphene floating on the etching solution. The graphene retained its square shape. Each TEM grid was then carefully transferred onto the surface of double-distilled water. This process was repeated at least twice to prevent contamination of the grid by the etching solution. After removing excess water with filter paper, the TEM grid was held by tweezers with the graphene-transferred side upward. A droplet of double-distilled water was carefully put on the grid and immediately sealed with a second TEM grid onto which graphene had been transferred in the same way, and which had been cut to form a semicircular shape for ease of contact with the lower TEM grid. Excess water was removed with filter paper. Within 30 min, the fabricated setup with the TEM grid was loaded into the TEM chamber. The resulting grid corresponds to that shown in Figure 1d.

### Fabrication of GLCs and GSLCs by the “Free-Standing” Method

For the fabrication of GLCs and GSLCs with free-standing graphene, the flattened graphene (6–8 layers CVD on a Cu foil; ACS Material, LLC; Pasadena, CA) was directly floated onto the etching solution, so that the Cu foil was in contact with the liquid surface. After treatment overnight, the Cu foil was completely etched, and the graphene that floated on the surface of the etching solution retained its square shape.

To fabricate the GLCs and GSLCs encapsulating pure water (Supporting Information; Figure S1b,c), the etching solution under floating graphene was carefully displaced with double-distilled water by using a pipet. This operation was repeated at least five times to minimize contamination by the etching solution. Then, the graphene floating on the double-distilled water was directly scooped with the graphene-transferred holey carbon TEM grid (Supporting Information; Figure S1b) or a SiN_*x*_ microwell TEM grid (Supporting Information; Figure S1c). After removal of excess water, the fabricated TEM grid was inserted into a TEM chamber within 30 min. The resulting holey carbon TEM grid and SiN_*x*_ microwell TEM correspond to those shown in Figures 2 and 3, respectively.

To fabricate the GSLCs encapsulating the etching solution (Supporting Information; Figure S1d), the graphene floating on the etching solution was directly scooped by the hydrophilically pretreated SiN_*x*_ microwell TEM grid, without replacement by double-distilled water. After removal of excess solution, the fabricated TEM grid was inserted into a TEM chamber within 30 min. The resulting grid corresponds to that shown in Figure 4a.

To fabricate the GSLCs encapsulating the aqueous (NH_4_)_2_SO_4_ solution (Supporting Information; Figure S1e), the etching solution under the floating graphene was carefully displaced with 0.4 M aqueous (NH_4_)_2_SO_4_ solution by using a pipet. The floating graphene was then scooped by the hydrophilically pretreated SiN_*x*_ microwell TEM grid. After removal of excess solution, the fabricated TEM grid was inserted into a TEM chamber within 30 min. The resulting grid corresponds to that shown in Figures 4b and 6a.

### Fabrication of Dried Highly Concentrated Aqueous Solutions

The target solution (1% w/v aqueous (NH_4_)_2_S_2_O_8_ solution or 0.4 M aqueous (NH_4_)_2_SO_4_ solution) was dropped onto a hydrophilically pretreated SiN_*x*_ microwell TEM grid. Excess solution was removed with filter paper. Within 30 min, the fabricated setup with the TEM grid was loaded into the TEM chamber after pre-evacuation.

### TEM Observation and Analysis

A JEM-2100F instrument (JEOL Ltd., Tokyo) was used for the TEM observations of the fabricated GLCs and GSLCs. An acceleration voltage of 200 kV was used for this study, except for the cryogenic experiment (Figure 2a,b), for which an acceleration voltage of 80 kV was selected. The TEM images were acquired by using a CMOS camera (OneView IS; Gatan, Inc., Pleasanton, CA). The highest count value of transmitted electrons in the initial frame of the video was used to calculate the dose rate of the Supporting Information Video S1. STEM–EDS spectra and images were acquired with a JED-2300T instrument (JEOL Ltd., Tokyo, Japan). ReciPro software^48^ was used for the SAED analysis. Standard diffraction data were taken from the American Mineralogist Crystal Structure Database (AMCSD). ImageJ software^49^ was used for the analysis of the bubbles. A liquid-helium-cooled TEM holder (Gatan, Inc., Pleasanton, CA) was used for the cryogenic experiment (Figure 2a,b). Liquid nitrogen was added to the holder, and the GLCs were cooled to 80 K. A Peltier cooling TEM holder (Mel-Build Co., Fukuoka) was used for the cooling experiment at 253.8 K (Figure 3g–i). The temperature was a calibrated value from a set value of 233.15 K. The actual value was higher than the set value due to the low heat conductivity of the holder to the GSLC assembly. Other observations of the GLCs and GSLCs were conducted at room temperature.

### SEM Observation of Etch Pits

To confirm the presence of etch pits in a Cu foil, 10 w/v % aqueous (NH_4_)_2_S_2_O_8_ solution was dropped onto the surface of 6–8 layers CVD graphene on Cu foil (ACS Material, LLC; Pasadena, CA). After 30 s, the foil was rinsed repeatedly with double-distilled water from a pipet. After the removal of excess water, the surface of the Cu foil was examined by SEM (TM4000Plus; Hitachi High-Tech Co., Tokyo). The secondary electron image was acquired at an acceleration voltage of 5 kV.

## Supporting Information Available

Fabrication methods for GLCs and GSLCs (Figure S1); Low-magnification bright-field TEM image of part of a TEM grid in which the formation of GLC was attempted by the conventional method. (Figure S2); A SiN_*x*_ microwell TEM grid used for the fabrication of GSLCs (Figure S3); STEM–EDS elemental mappings and corresponding spectrum of the GSLC shown in Figure 3g (Figure S4); Movement of bubbles in GSLCs (Figure S5); Enlarged snapshots of the bubble in the GSLC of the etching solution (Figures S6–S8); Enlarged snapshots of the bubble in the GSLC of the 0.4 M aqueous (NH_4_)_2_SO_4_ solution (Figure S9); STEM–EDS elemental mappings and the corresponding spectrum of the etching solution in an open system (Figure S10); Evaporation of 0.4 M aqueous (NH_4_)_2_SO_4_ solution in an open system (Figure S11); The presence of graphene around and on a GSLC (Figure S12); and Leakage of water from a GSLC in air without electron-beam irradiation (Figure S13) (PDF)
Video S1: Bubble movements in a GLC in folded graphene encapsulating displaced water from the etching solution (MP4)
Video S2: Bubble movements in a GSLC encapsulating displaced water from the etching solution (MP4)
Video S3: A micrometer-scale GSLC encapsulating the etching solution (MP4)
Video S4: A submicrometer-scale GSLC encapsulating 0.4 M aqueous (NH_4_)_2_SO_4_ solution (MP4)
Video S5: Sublimation of the dried ten-times-diluted etching solution with bubbling (MP4)
Video S6: Liquid-like behavior of the dried 0.4 M aqueous (NH_4_)_2_SO_4_ solution with deformation and coalescence of bubbles (MP4)
Video S7: Decreasing area of a bubble in a GSLC of 0.4 M aqueous (NH_4_)_2_SO_4_ solution (MP4)
