# Supplementary material for: Micrometer-Scale Graphene-Based Liquid Cells of Highly Concentrated Salt Solutions for In Situ Liquid-Cell Transmission Electron Microscopy
Source: ACS Omega. 2024 Sep 12;9(38):39914–24. doi: 10.1021/acsomega.4c05477 (PMC11425617; doi:10.1021/acsomega.4c05477)
Supplement: Supplementary file 1 — ao4c05477_si_001.pdf [file ao4c05477_si_001.pdf]

Supporting Information

Micrometer-scale graphene-based liquid cells of highly concentrated salt solutions for in situ liquid-cell transmission electron microscopy

Yuga Yashima, Tomoya Yamazaki, & Yuki Kimura
Institute of Low Temperature Science, Hokkaido University, Sapporo, Japan
Email for the corresponding author: ykimura@lowtem.hokudai.ac.jp

Supporting Information Video S1

Bubble movements in a GLC in folded graphene encapsulating displaced water from the etching solution. The time and scale are presented in the video. Dose rate is 166 $e^- Å^{-2} s^{-1}$. Frames of the video at 0.00, 3.00 and 6.00 s are presented in Figure 2c.

Supporting Information Video S2

Bubble movements in a GSLC encapsulating displaced water from the etching solution. The time and scale are presented in the video. Dose rate is 48 $e^- Å^{-2} s^{-1}$. Frames of the video at 5.00, 10.00 and 15.00 s are presented in Figures 3d-f.

Supporting Information Video S3

A micrometer-scale GSLC encapsulating the etching solution. The time and scale are presented in the video. Dose rate is 1 $e^- Å^{-2} s^{-1}$. Frames of the video at 0, 10 and 20 s are presented in Figure 4a. A detailed analysis of the bubbles in the GSLC is presented in Supporting Information Figures S5–S8.

Supporting Information Video S4

A submicrometer-scale GSLC encapsulating 0.4 M aqueous $(NH_4)_2SO_4$ solution. The time and scale are presented in the video. Dose rate is 28 $e^- Å^{-2} s^{-1}$. Frames of the video at 0, 10 and 20 s are presented in Figure 4b. A detailed analysis of the bubbles in the GSLC is presented in Supporting Information Figures S5 and S9.

Supporting Information Video S5

Sublimation of the dried ten-times-diluted etching solution with bubbling. The time and scale are presented in the video. Dose rate is 1 $e^- Å^{-2} s^{-1}$. Frames of the video at 23.00, 33.00 and 43.00 s are presented in Figure 5a.

Supporting Information Video S6

Liquid-like behavior of the dried 0.4 M aqueous $(NH_4)_2SO_4$ solution with deformation and coalescence of bubbles. The time and scale are presented in the video. Dose rate is 48 $e^- Å^{-2} s^{-1}$. Frames of the video at 18.00, 26.00 and 34.00 s are presented in Figure 5b.

Supporting Information Video S7

Decreasing area of a bubble in a GSLC of 0.4 M aqueous $(NH_4)_2SO_4$ solution. The time and scale are presented in the video. Dose rate is 186 $e^- Å^{-2} s^{-1}$. Frames of the video at 0.00 and 60.00 s are presented in Figure 6a. Enlarged views of the inner droplets of the large bubble at 35.00 and 55.00 s are presented in Figure 6d.

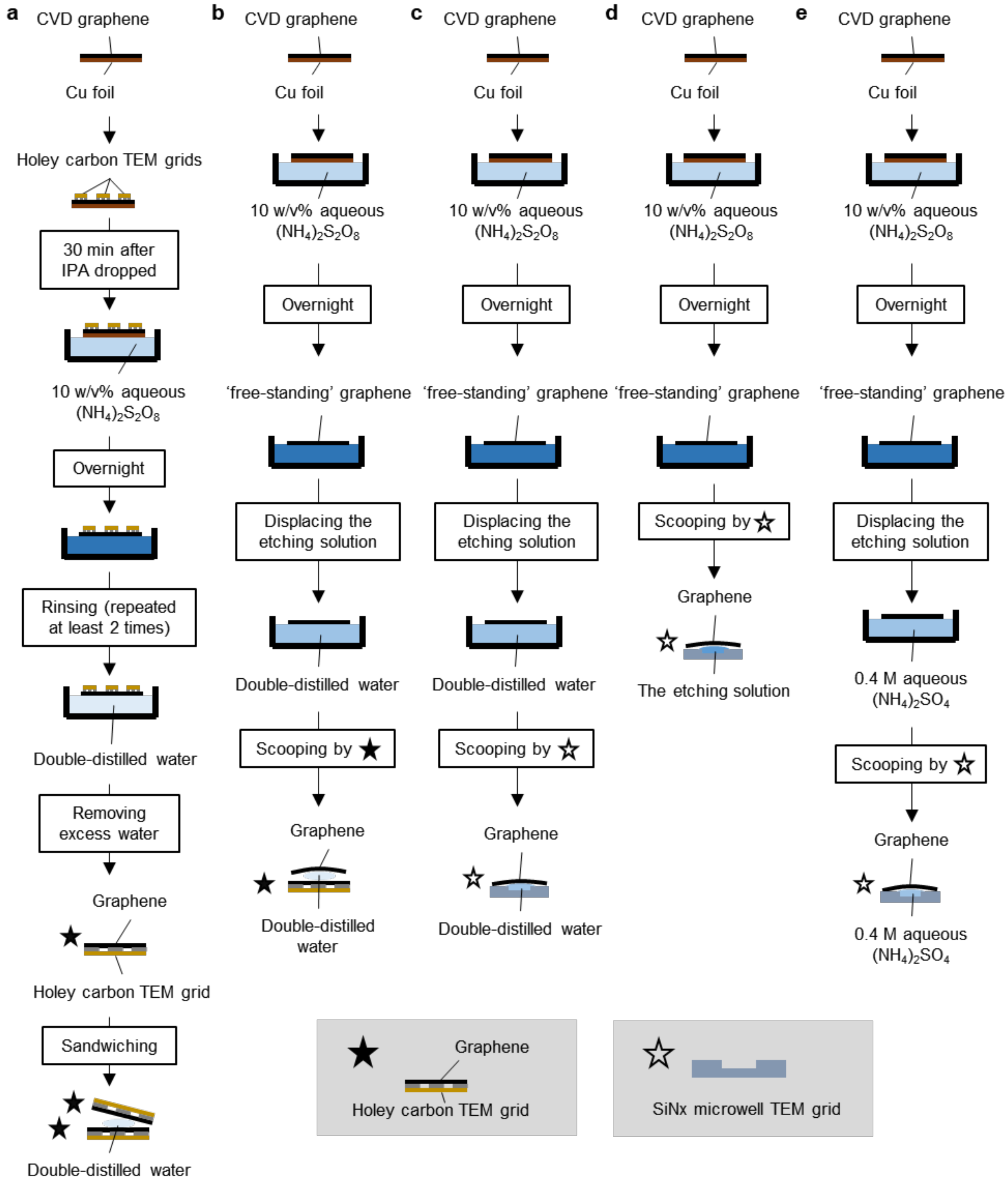

Figure S1. Fabrication methods for GLCs and GSLCs. **a**, The conventional fabrication method for GLC encapsulation of pure water with two graphene-transferred holey carbon TEM grids. The resulting grid corresponds to that in Figure 1d. **b**, The free-standing fabrication method for GLCs encapsulating pure water with a free-standing graphene sheet on the liquid surface. The resulting grid corresponds to that in Figure 2. **c**, The free-standing fabrication method for GSLCs encapsulating an etching solution with a free-standing graphene sheet on the liquid surface. The resulting grid corresponds to that in Figure 3. **d**, The free-standing fabrication method for GSLCs encapsulating the etching solution with a free-standing graphene sheet on the liquid surface. The resulting grid corresponds to that in Figure 4a. **e**, The free-standing fabrication method for GSLCs encapsulating aqueous $(NH_4)_2SO_4$ with a free-standing graphene sheet on the liquid surface. The resulting grid corresponds to that in Figures 4b and 6a.

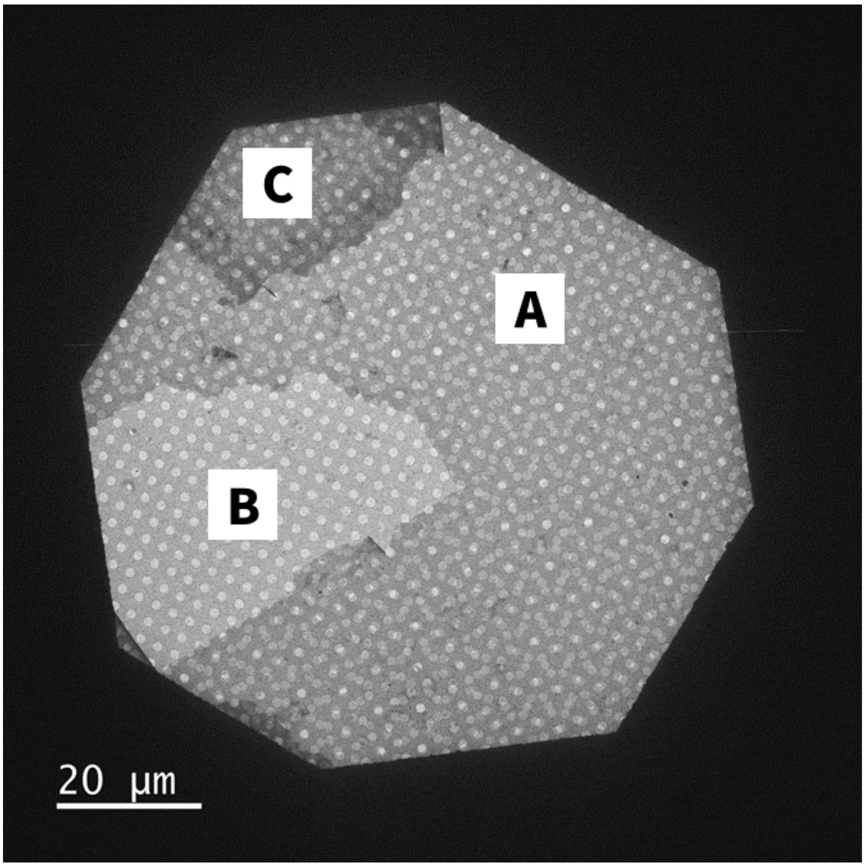

Figure S2. Low-magnification bright-field TEM image of part of a TEM grid in which the formation of GLC was attempted by the conventional method. The grid was fabricated by placing two TEM grids with graphene sheets facing each other and attempting to sandwich droplets of pure water (Supporting Information; Figure S1a). Two sheets of graphene-transferred holey carbon sheets faced each other in the area A. A carbon sheet on one side was partially broken, resulting in the brighter contrast in the area B. In the darker contrast area C, there was another stack of a graphene-transferred carbon sheet on the two sheets, presumably this was a piece that ruptured in the area B. No contrast gradations or bubbles over the entire field of view were observed, only contrast changes with the number of carbon sheets and contrast due to contamination.

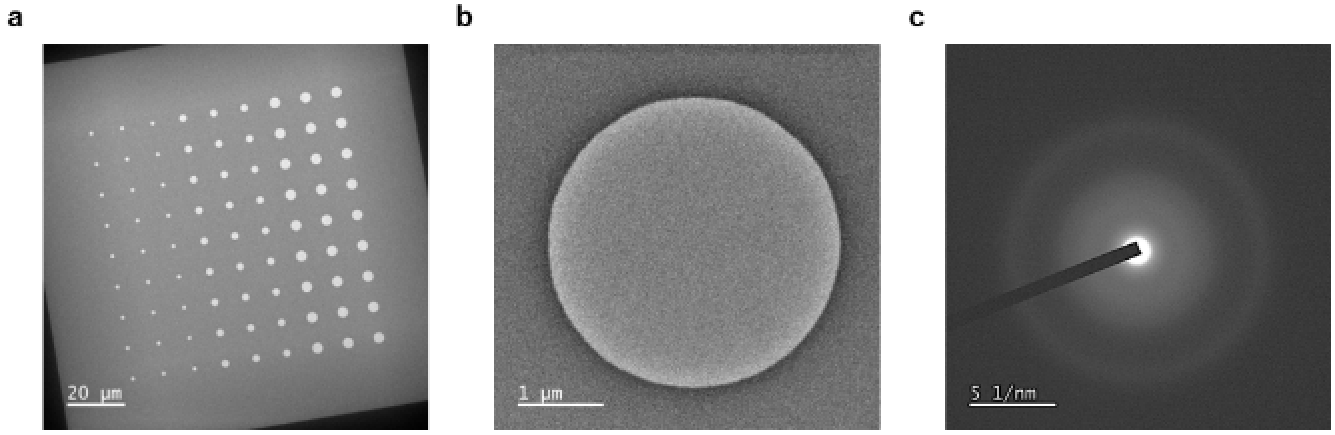

Figure S3. A SiN_x microwell TEM grid used for the fabrication of GSLCs**. a**, A bright-field TEM image of one of the windows of the SiN_x microwell TEM grid. In each window, there are 81 microwells (9 × 9) with three different diameters (1, 2, and 3 µm). Each microwell is 60 nm deep between the SiN_x membranes of thickness 100 and 40 nm. **b**, Enlarged view of a 3-µm-diameter microwell. The thickness in the circular area is 40 nm and that of the surrounding area is 100 nm. **c**, SAED pattern acquired from the circular area of **b**.

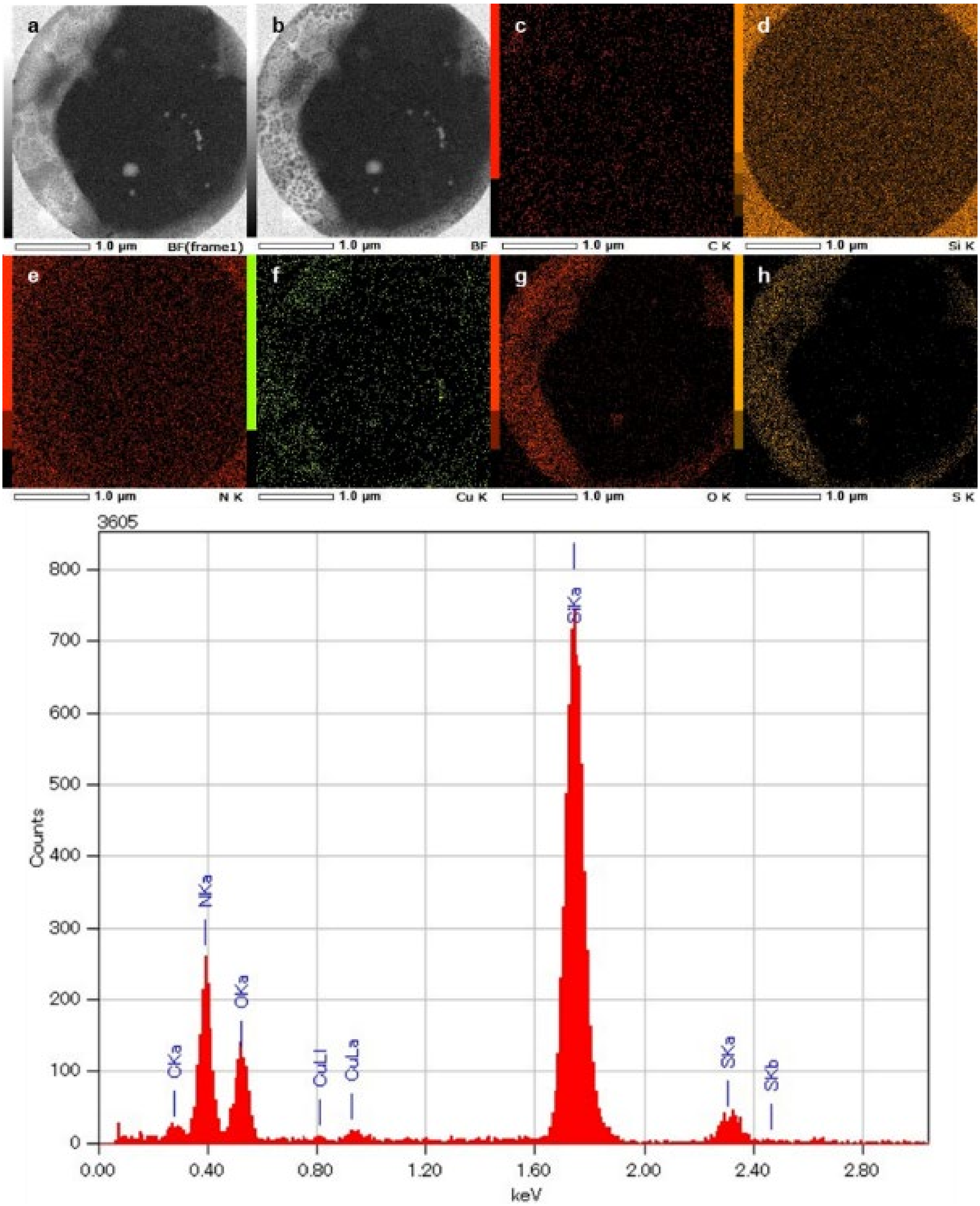

Figure S4. STEM-EDS elemental mappings and corresponding spectrum of the GSLC shown in Figure 3g. The GSLC was fabricated by scooping free-standing graphene floated on water used to displace the etching solution (Supporting Information; Figure S1c). The GSLC was cooled at 253.8 K. Each panel is high-angle annular dark-field scanning TEM (HAADF−STEM) image; **a** before accumulation of the data, **b** after accumulation the data, and the corresponding elemental mappings for **c**, carbon, **d**, silicon, **e**, nitrogen, **f**, copper **g**, oxygen, and **h**, sulfur. Each mapping is rotated 20° counterclockwise relative to Figure 3g.

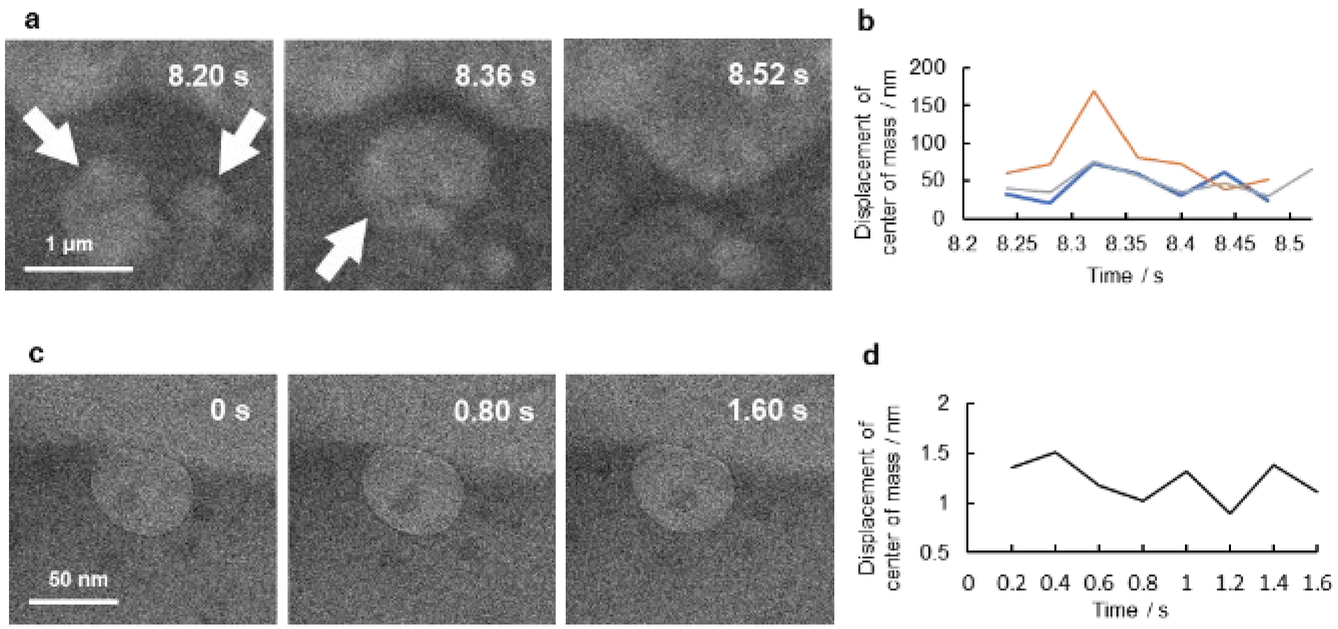

Figure S5. Movement of bubbles in GSLCs. **a**, Series of bright-field TEM images of a bubble in the middle of the GSLC of the etching solution. The full video is available in Supporting Information Video S3. The bubbles (white arrows) at 8.20 s coalesced by 8.36 s (white arrow) and merged into the larger bubble above at 8.52 s. These snapshots correspond to the central part in Figure 4a and Supporting Information Video S3. Detail snapshots are shown in Supporting Information Figure S6. **b**, Temporal evolution of the displacement of the center of mass per 0.04 s of the bubble in **a** (blue line). The orange and gray lines are from two other bubbles shown in Supporting Information Figures S7 and S8. Calculated velocities of the centers of mass were 1.08, 1.96, and 1.21 μm s^{-1} for the blue, orange, and gray lines, respectively. The average velocity of the three bubbles was 1.42 μm s^{-1}. **c**, Series of bright-field TEM images of a bubble in the GSLC of the 0.4 M aqueous $(NH_4)_2SO_4$ solution at 0.00, 0.80, and 1.60 s; the same bubble is indicated by a white arrow in Figure 4b. Detail snapshots are shown in Supporting Information Figure S9. The full video is available in Supporting Information Video S4. **d**, Temporal evolution of the displacement of the center of mass per 0.20 s of the bubble in **c**. The velocity of the center of mass of the bubble was calculated to be 6.10 nm s^{-1}. This was significantly smaller than that in **a**. The more than two orders of magnitude difference in the velocities of the bubbles might be due to such factors as differences in the geometry of the GSLCs and differences in the thickness of the encapsulating liquid. More bending of the graphene would be facilitated in the larger GSLC of **a**, especially in the middle area, due to much radiolysis and subsequent bubbling. This could lead to a more flexible geometry for the bubbles themselves, permitting more-dynamic bubble movement compared with that of **b**.

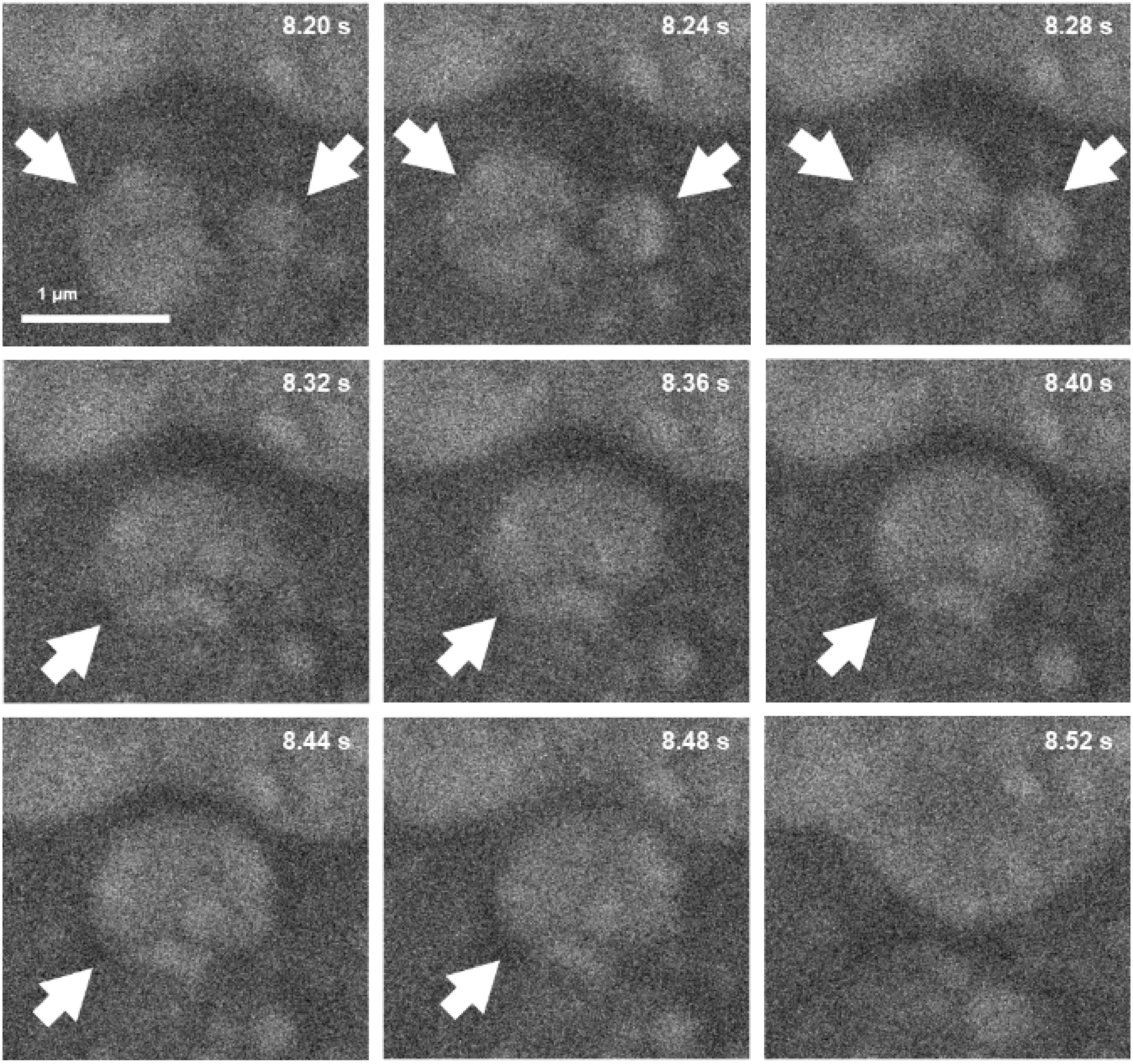

Figure S6. Enlarged snapshots of the bubble in the GSLC of the etching solution. The GSLC was fabricated by encapsulating the etching solution on a SiN_x microwell grid with free-standing graphene (Supporting Information; Figure S1d). The bubbles used in the calculation of the displacement of mass center as shown by the blue line in Supporting Information Figure S5b are indicated by white arrows. There were two bubbles in each snapshot at 8.20, 8.24, and 8.28 s. Before the coalescence, the mass centers of the two bubbles were used for the tracking. The snapshots at 8.20, 8.36, and 8.52 s are also shown in Supporting Information Figure S5a. The full video is available in Supporting Information Video S3.

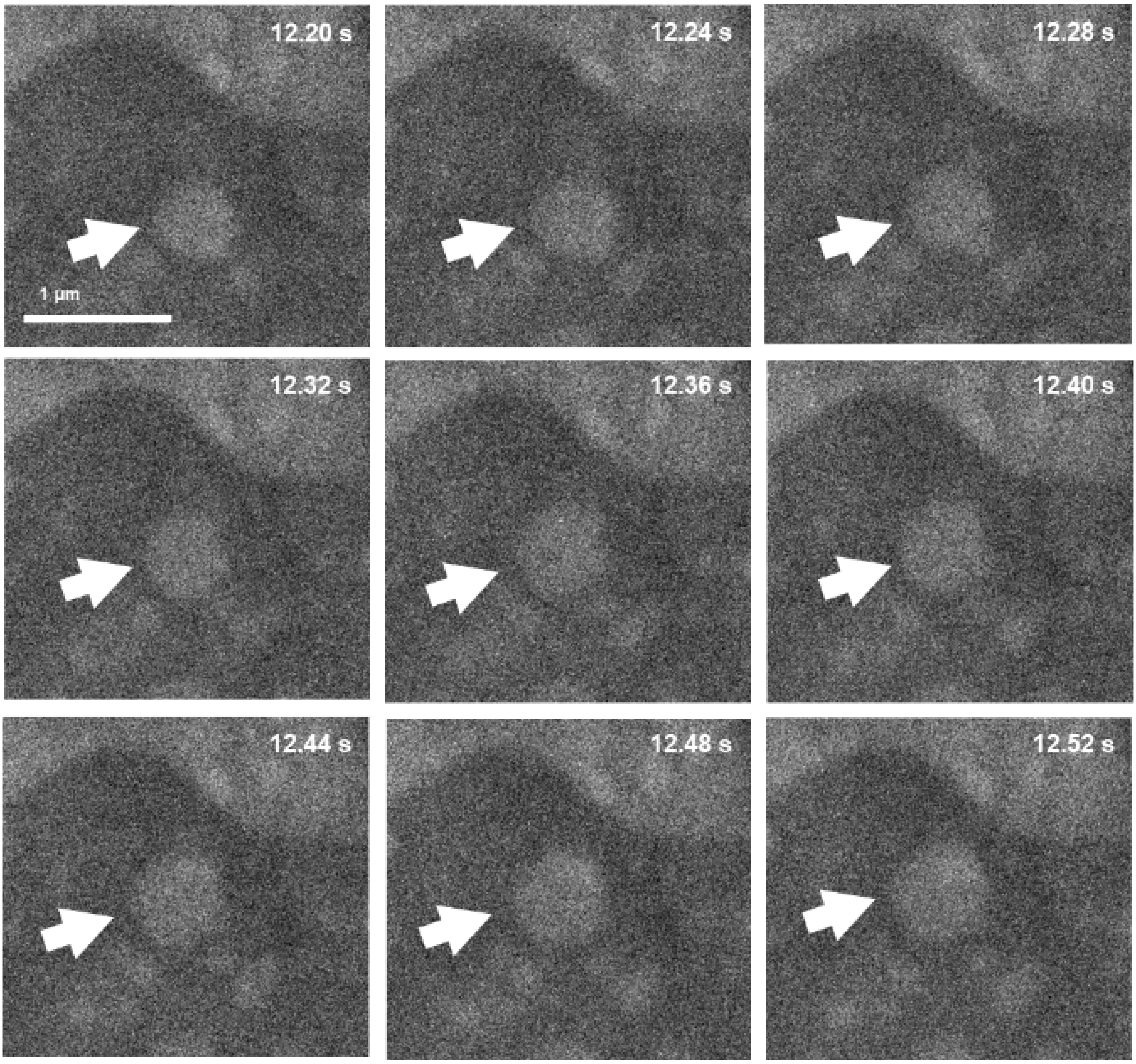

Figure S7. Enlarged snapshots of the bubble in the GSLC of the etching solution. The GSLC was fabricated by encapsulating the etching solution on a SiN_x microwell grid with free-standing graphene (Supporting Information; Figure S1d). The bubbles used in the calculation of the displacement of the mass center shown by the orange line in Supporting Information Figure S5b are indicated by white arrows. The full video is available in Supporting Information Video S3.

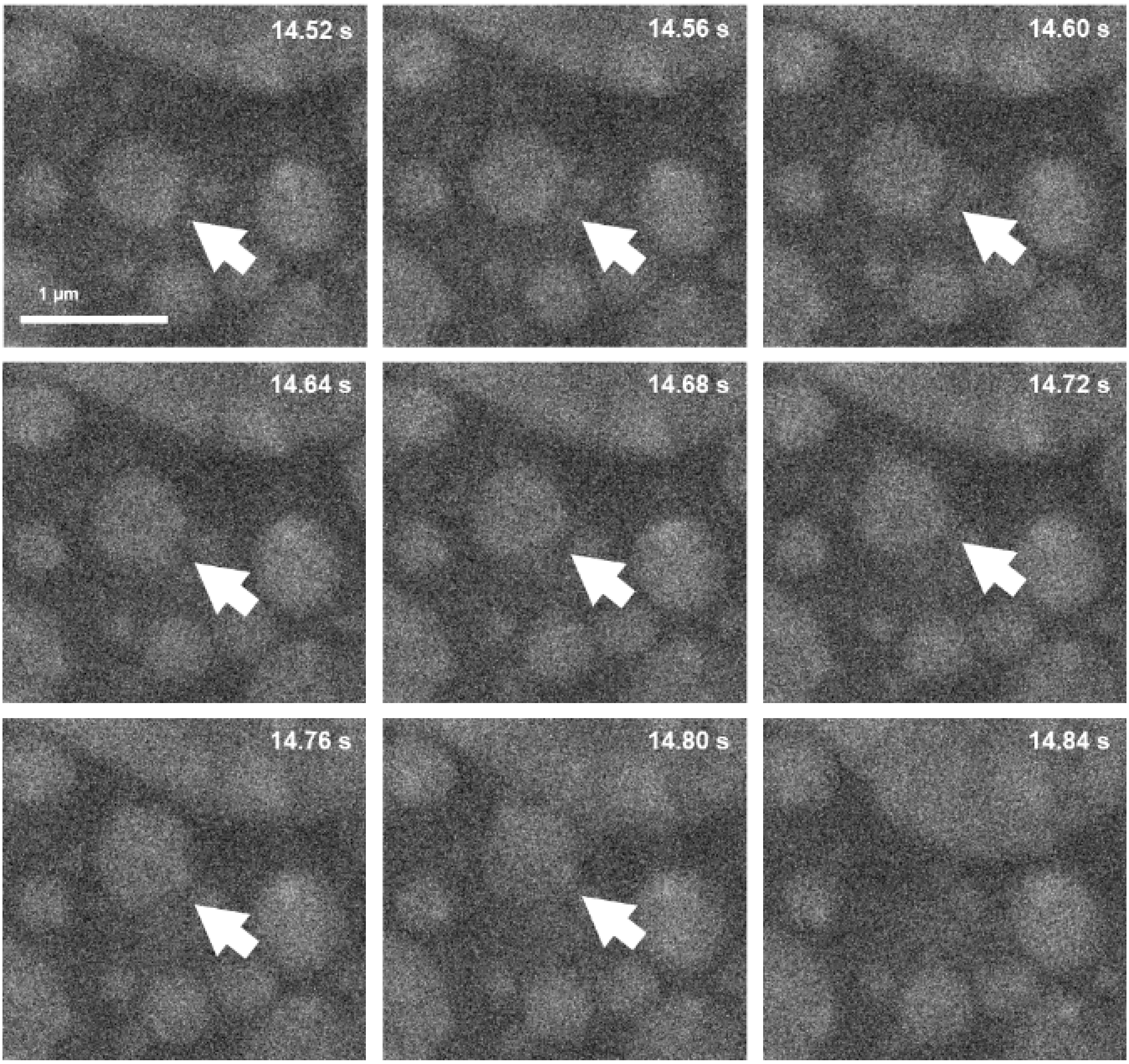

Figure S8. Enlarged snapshots of the bubble in the GSLC of the etching solution. The GSLC was fabricated by encapsulating the etching solution on a SiN_x microwell grid with free-standing graphene (Supporting Information; Figure S1d). The bubbles used in the calculation of the displacement of the mass center shown by the gray line in Supporting Information Figure S5b are indicated by white arrows. The full video is available in Supporting Information Video S3.

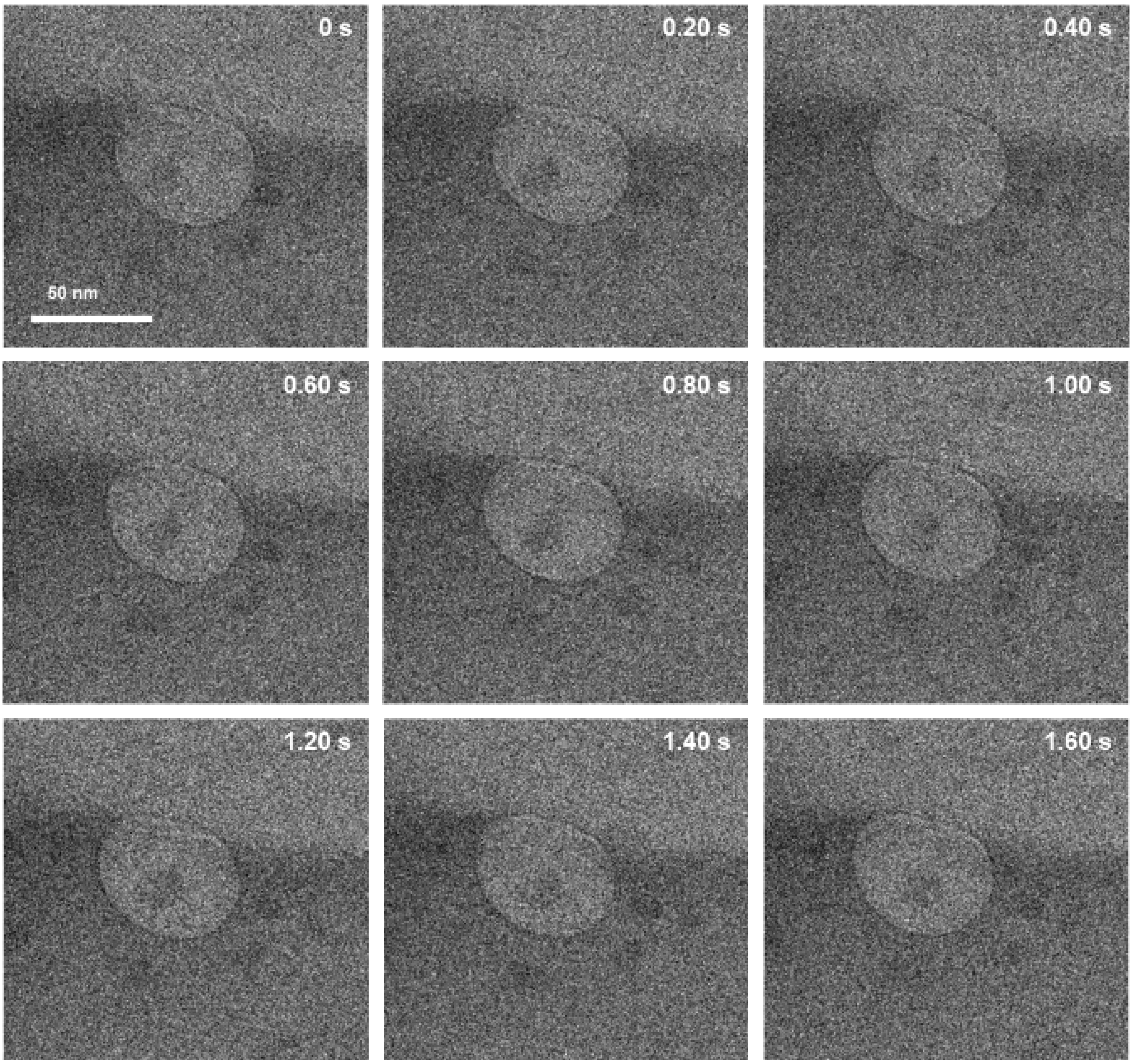

Figure S9. Enlarged snapshots of the bubble in the GSLC of the 0.4 M aqueous $(NH_4)_2SO_4$ solution. The GSLC was fabricated by encapsulating 0.4 M aqueous $(NH_4)_2SO_4$ solution on a SiN_x microwell TEM grid with free-standing graphene (Supporting Information; Figure S1e). The snapshots at 0.00, 0.80, and 1.60 s are also shown in Supporting Information Figure S5c. The temporal evolution of the displacement of the center of mass per 0.20 s of the bubble is shown in Supporting Information Figure S5d. The full video is available in Supporting Information Video S4.

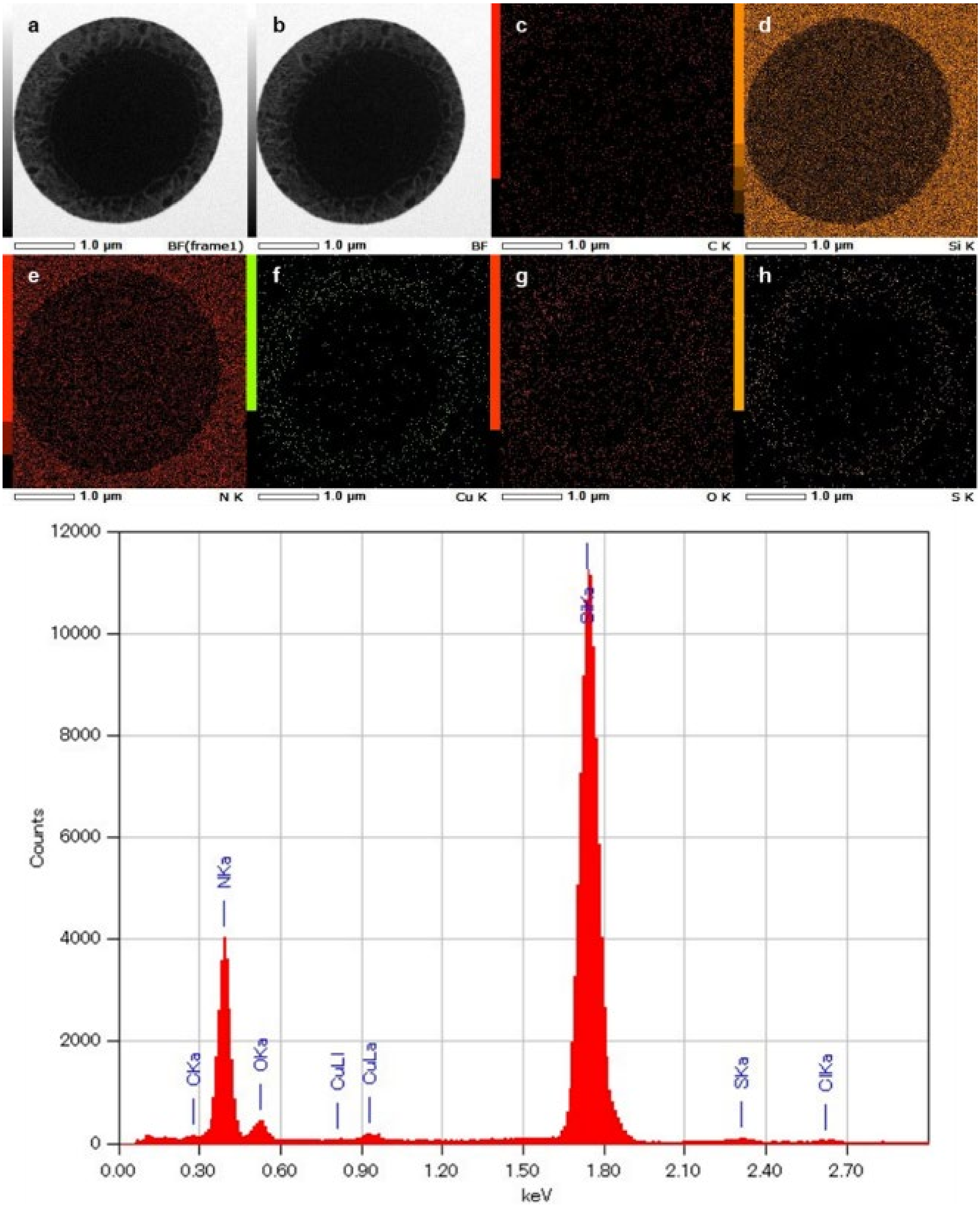

Figure S10. STEM-EDS elemental mappings and the corresponding spectrum of the etching solution in an open system. 1% w/v aqueous $(NH_4)_2S_2O_8$ solution was dropped onto a SiN_x microwell TEM grid. The mappings and corresponding spectrum were acquired just after the recording of Supporting Information Video S5 for the same microwell. Enlarged snapshots are shown in Figure 5a. Each panel is a HAADF-STEM image **a,** before accumulation the data **b**, after accumulation the data, and elemental mappings of **c**, carbon, **d**, silicon **e**, nitrogen **f**, copper **g**, oxygen, and **h**, sulfur.

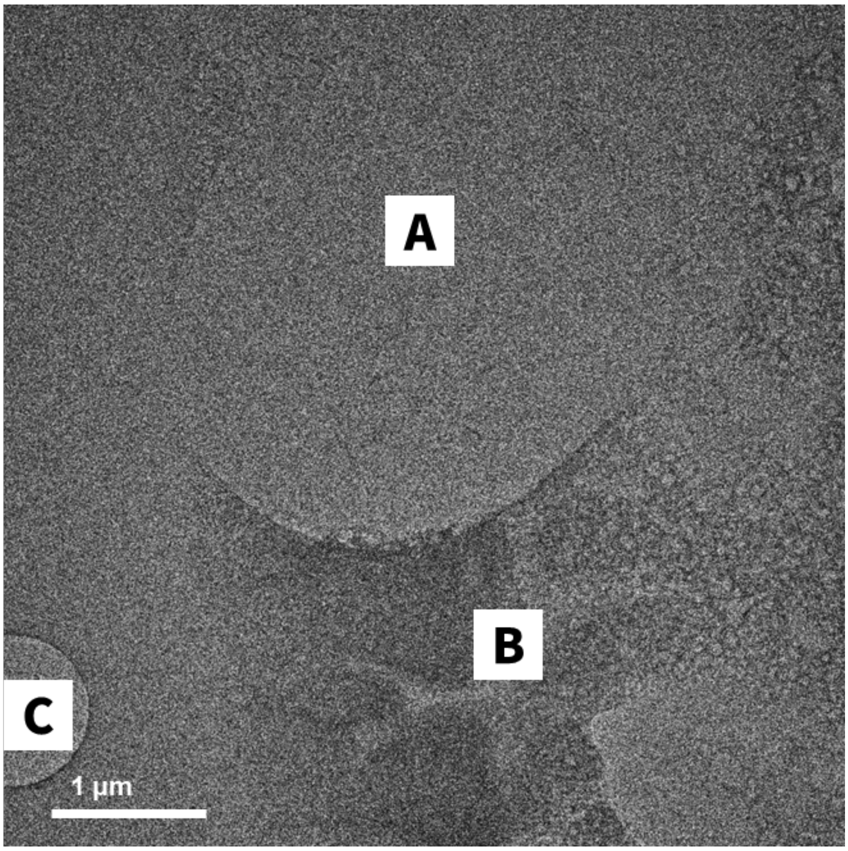

Figure S11. Bright field TEM image showing evaporation of 0.4 M aqueous $(NH_4)_2SO_4$ solution in an open system. A 0.4 M aqueous solution of $(NH_4)_2SO_4$ was simply dropped onto a SiN_x microwell TEM grid**.** This TEM image is a snapshot of Supporting Information Video S6 at 40 s. Bubbling and liquid-like behavior of the solution in the exposed area A at 18, 26, and 34 s is shown in Figure 5b. The solution was finally evaporated, then the exposed area A showed a brighter contrast compared to that of the less exposed area B. The area C is corresponding to a SiN_x microwell.

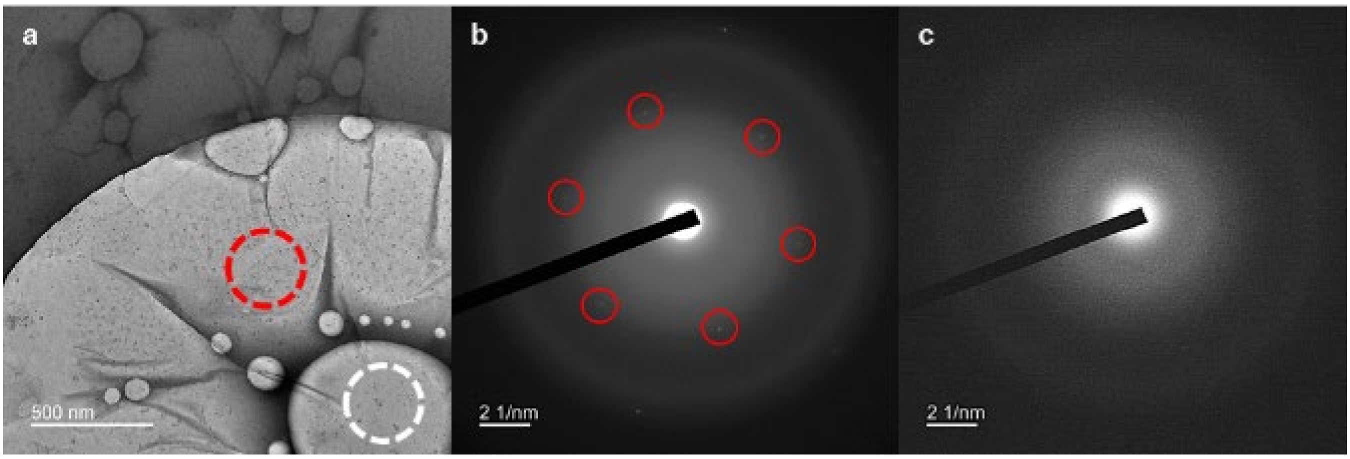

Figure S12. The presence of graphene around and on a GSLC. **a**, Bright-field TEM image of the GSLC encapsulating 0.4 M aqueous $(NH_4)_2SO_4$ solution on a SiN_x microwell grid, taken at 190 s for Figure 6a. **b**, SAED pattern obtained from red dotted circular area in **a**. The six-fold symmetric pattern is highlighted by the red circles. **c**, SAED pattern obtained from the center of the GSLC (the white dotted circle in **a**).

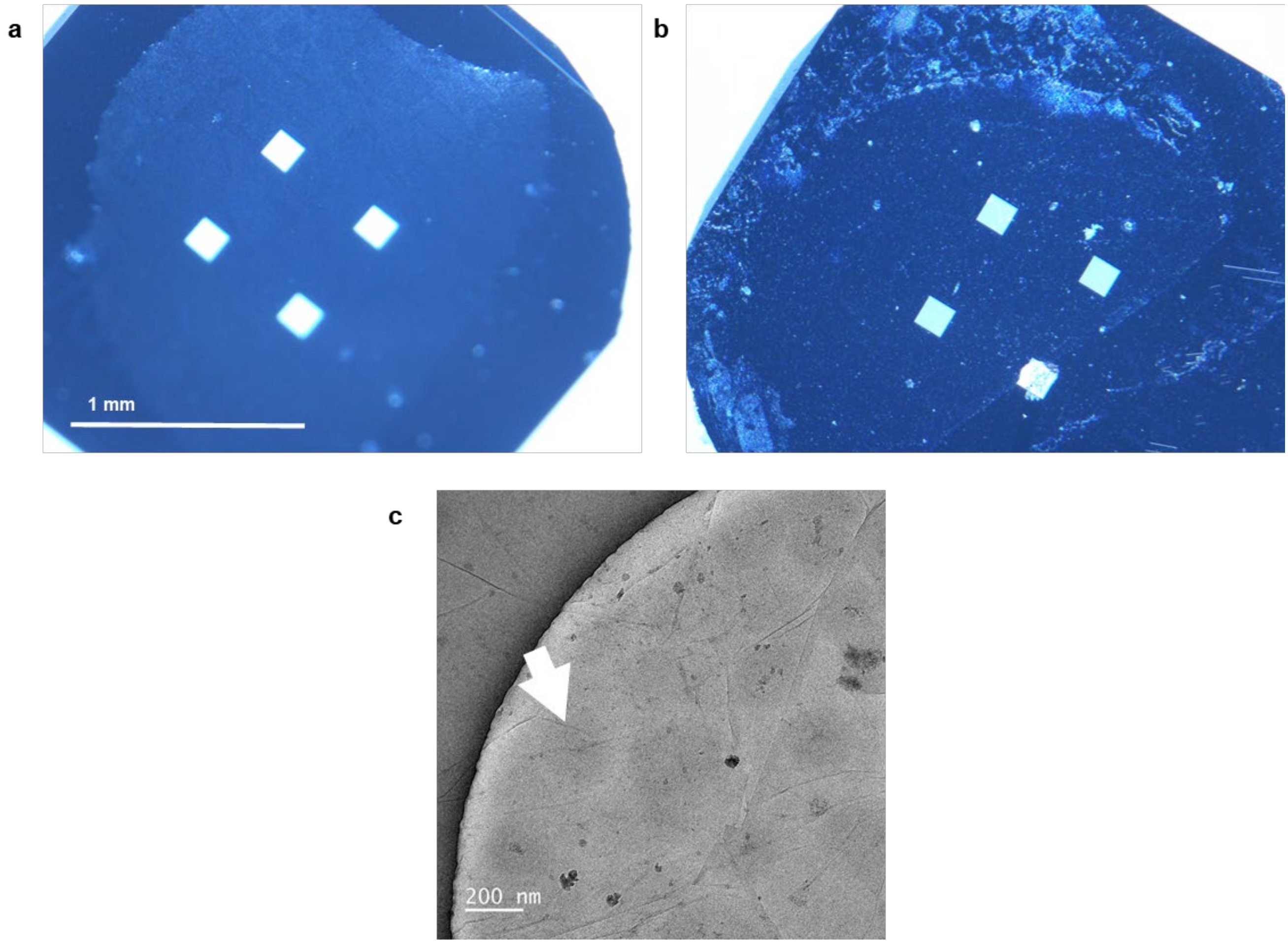

Figure S13. Leakage of water from a GSLC in air without electron-beam irradiation. **a**, Optical image of a SiN_x microwell TEM grid encapsulating water supercooled to −5 °C. **b**, Optical image of the SiN_x microwell TEM grid after being kept overnight in a freezer at −5 °C. **c**, Bright-field TEM image of a microwell edge in **b**. One of the circular dark contrasts is indicated by a white arrow.
